# Cost-effectiveness of a structured medication review approach for multimorbid older adults: Within-trial analysis of the OPERAM study

**DOI:** 10.1371/journal.pone.0265507

**Published:** 2022-04-11

**Authors:** Paola Salari, Cian O’Mahony, Séverine Henrard, Paco Welsing, Arjun Bhadhuri, Nadine Schur, Marie Roumet, Shanthi Beglinger, Thomas Beck, Katharina Tabea Jungo, Stephen Byrne, Stefanie Hossmann, Wilma Knol, Denis O’Mahony, Anne Spinewine, Nicolas Rodondi, Matthias Schwenkglenks

**Affiliations:** 1 Institute of Pharmaceutical Medicine (ECPM), University of Basel, Basel, Switzerland; 2 Pharmaceutical Care Research Group, School of Pharmacy, University College Cork, Cork University Hospital, Cork, Ireland; 3 Louvain Drug Research Institute, Clinical Pharmacy Research Group, UCLouvain, Brussels, Belgium; 4 Institute of Health and Society (IRSS), UCLouvain, Brussels, Belgium; 5 Division of Internal Medicine and Dermatology, University Medical Centre Utrecht, Utrecht, The Netherlands; 6 Clinical Trial Unit Bern, University of Bern, Bern, Switzerland; 7 Department of General Internal Medicine, Inselspital, Bern University Hospital, University of Bern, Bern, Switzerland; 8 Institute of Primary Health Care (BIHAM), University of Bern, Bern, Switzerland; 9 Department of Geriatric Medicine and Expertise Centre Pharmacotherapy in Old Persons, University Medical Centre Utrecht, Utrecht University, Utrecht, The Netherlands; 10 Department of Medicine (Geriatrics), University College Cork, Cork University Hospital, Cork, Ireland; 11 Pharmacy Department, CHU UCL Namur, Yvoir, Belgium; National Center for Global Health and Medicine, JAPAN

## Abstract

**Background:**

Inappropriate polypharmacy has been linked with adverse outcomes in older, multimorbid adults. OPERAM is a European cluster-randomized trial aimed at testing the effect of a structured pharmacotherapy optimization intervention on preventable drug-related hospital admissions in multimorbid adults with polypharmacy aged 70 years or older. Clinical results of the trial showed a pattern of reduced drug-related hospital admissions, but without statistical significance. In this study we assessed the cost-effectiveness of the pharmacotherapy optimisation intervention.

**Methods:**

We performed a pre-planned within-trial cost-effectiveness analysis (CEA) of the OPERAM intervention, from a healthcare system perspective. All data were collected within the trial apart from unit costs. QALYs were computed by applying the crosswalk German valuation algorithm to EQ-5D-5L-based quality of life data. Considering the clustered structure of the data and between-country heterogeneity, we applied Generalized Structural Equation Models (GSEMs) on a multiple imputed sample to estimate costs and QALYs. We also performed analyses by country and subgroup analyses by patient and morbidity characteristics.

**Results:**

Trial-wide, the intervention was numerically dominant, with a potential cost-saving of CHF 3’588 (95% confidence interval (CI): -7’716; 540) and gain of 0.025 QALYs (CI: -0.002; 0.052) per patient. Robustness analyses confirmed the validity of the GSEM model. Subgroup analyses suggested stronger effects in people at higher risk.

**Conclusion:**

We observed a pattern towards dominance, potentially resulting from an accumulation of multiple small positive intervention effects. Our methodological approaches may inform other CEAs of multi-country, cluster-randomized trials facing presence of missing values and heterogeneity between centres/countries.

## Introduction

A large proportion of current healthcare spending in developed countries is on multimorbid older adults. A significant proportion of healthcare funds may be wasted on overtreatment i.e. unnecessary interventions and inappropriate medications. Inappropriate medications in older adults may trigger adverse events, such as bleeding, falls and fractures, resulting in drug-related hospital admissions (DRAs) [1], which are costly and potentially preventable [2, 3]. Few studies have assessed the costs and effectiveness of medication-optimisation interventions, and furthermore the evidence from these studies is mixed. Some evidence of reduced DRAs was found for hospital visits, emergency department visits and drug-related readmissions [4]. Two recent Cochrane reviews, which evaluated interventions to optimise prescribing for older adults in nursing homes, found that the effect of these interventions on drug costs was unclear [5]. Another review on pharmacist-participated medication management for older adults in nursing homes found favourable economic outcomes, albeit not all statistically significant [6]. Similarly, one study focussed on an intervention to reduce potentially inappropriate prescribing for older people found uncertainty with respect to its cost-effectiveness [7].

The OPERAM (*OPtimising thERapy to prevent Avoidable hospital admissions in the Multimorbid older people*) trial (ClinicalTrials.gov Identifiers: main trial: NCT02986425; health economic sub-study: NCT03108092) was a cluster-randomized trial conducted between 2016 and 2019, aimed at reducing preventable DRAs (primary endpoint) in adults aged 70 years or older with multi-morbidity (i.e. ≥ 3 coexistent chronic conditions defined by ICD-10 codes) and polypharmacy (i.e. ≥ 5 different regular drugs for more than 30 days), through improving pharmacotherapy [8, 9].

The trial found no statistically significant clinical effect on its primary endpoint [10]. However, there were multiple, small intervention effects, consistently in the expected direction (e.g. the hazard ratio for DRA was 0.87 and for death 0.90 when restricting intervention patients to those who had ≥1 potentially inappropriate medication discontinued at 2 months). Hence, it remained important to assess the effect of the trial intervention on (general) health relative to its economic impact.

We performed a pre-planned, within-trial cost-effectiveness analysis (CEA) of the OPERAM trial. This involved a series of methodological challenges, principally the clustered structure of the trial data, possible heterogeneity between centres/countries and the unavoidable occurrence of missing data. Published literature offers abundant guidance on such methodological issues arising in within-trial cost-effectiveness studies [11–13]. However, these are usually treated in isolation and there is very little guidance on how they should be dealt with in combination. The methodological approach applied in our analysis combined all these aspects.

## Materials and methods

### Design of the OPERAM trial

The overall objective of the OPERAM trial was to assess whether a software-assisted approach to pharmacotherapy optimisation, namely the *Systematic Tool to Reduce Inappropriate Prescribing* (STRIP) based on STOPP/START criteria [14] and including STRIP assistant (STRIPA), implemented by an interprofessional team composed of a medical doctor and a pharmacist, led to an improvement in clinical and economic outcomes in the target population, enrolled at the beginning of an index acute non-specific hospitalisation episode [15]. The trial was cluster-randomised with clusters defined by a prescribing hospital physician. The control group included patients receiving usual care. The trial was performed in four clinical centres in Europe, namely University Hospital Bern, Switzerland; Saint-Luc University Hospital, Belgium; Cork University Hospital, Cork, Ireland; and University Medical Centre Utrecht, The Netherlands. The planned sample size was 2,000 patients, enrolled in 80 clusters. Each cluster represented a group of patients treated by the same consultant in the same ward. After their initial hospitalization, patients were followed up by telephone interviews at 2, 6 and 12 months from randomization. More details are available from the trial protocol [8, 9] and trial publication [10].

### Approach to cost-effectiveness analysis

We followed a detailed health economic analysis plan (HEAP) developed before the end of the trial and last modified before the lock of the trial database (for deviations from the HEAP, see S1 Section in S1 File). All health economic data elements, including those covering medical resource use and utilities, were collected within the trial, with the exception of unit cost data. Cost-effectiveness was estimated for a one-year time horizon, aligned with the 12 month follow-up period of the OPERAM trial. Due to the one-year time horizon, discounting was not applied. We adopted a healthcare system perspective based on local unit costs for the main analysis, with cost results expressed in Swiss francs (CHF), as Switzerland was the largest recruiter into the OPERAM trial. We also approximated a societal perspective for a secondary analysis, by adding the costs of informal care. Given the clustered nature of the trial data, together with the likely presence of between-country heterogeneity, we adopted a regression-based approach [13]. We applied Generalized Structural Equation Models (GSEMs) that allow simultaneous estimation of costs and QALYs, and in the process account for the clustered structure of the data (i.e. the clusters were treated as random effects) [16]. Individual patient characteristics and country fixed effects were added to the models, the former as potential confounders given residual baseline imbalances, the latter to account for between-centre/country heterogeneity [17–19]. We assessed heterogeneity between centres/countries using the method described by Cook et al., by testing for qualitative and quantitative interaction on the key outcomes of incremental QALYs and incremental costs [20, 21].

### Calculation of quality-adjusted life years

Information on utilities was collected using the European Quality of Life-5 Dimensions (EQ-5D-5L) instrument [22–26]. We combined the EQ-5D-5L responses with the interim (crosswalk) valuation algorithm for Germany to calculate utility scores [27–29], to approximate a Swiss perspective given the absence of a Swiss valuation algorithm [30]. In the country-specific analyses, we applied the German crosswalk algorithm for Switzerland, the UK crosswalk algorithm for Ireland, and the Dutch crosswalk algorithm for Belgium and the Netherlands. QALYs were estimated for the one-year trial follow-up period, using standard area under the curve methods following the trapezium rule [21]. For patients who died during the trial, we set utility to zero from the date of death.

### Calculation of costs

Based on unit cost data from non-trial sources, the following cost items were included in the main analysis: costs of hospitalizations, rehabilitation facilities, medical visits, nursing visits at home, nursing home care and drugs. Costs of informal care was included in a secondary analysis approximating a societal perspective. We applied local unit costs for the year 2018 to each country, similar to previous studies [13, 31–33]. We converted all local unit costs into one common currency (Swiss Francs, CHF), using purchasing power parities (PPP) [34], as recommended in the literature [13, 32, 33, 35].

Regarding sources of unit cost data, hospitalization costs were estimated using diagnosis-related group-based (DRGs) reimbursement for Switzerland [36] and Ireland [37], retrieved from validated hospital data for Belgium [38] and estimated using the Dutch manual for costing studies in healthcare for the Netherlands [39]. Costs of outpatient physician visits by specialty, and for visits with other healthcare providers (e.g. physiotherapists), were provided by a provider of statutory health insurance for Switzerland, taken from the National Institute for Health and Disability Insurance (NIHDI) website for Belgium [40], and based on national costing studies for the Netherlands [16] and Ireland [41, 42]. Costs of nursing visits at home, nursing home care and stays in rehabilitation facilities were drawn from national statistical data for Switzerland [43], Ireland [44] and Belgium [40] and from national costing studies for the Netherlands [16]. Drug costs were drawn from official data sources for Switzerland [45] and Belgium [46], adapted from Belgium for the Netherlands [47] and estimated using a pre-purchased wholesaler price list for Ireland. The costing of the STRIP intervention was based on estimated cost of the software (based on similar products available on the market), and trial-observed staff times and staff costs for all countries. Finally, costs of informal, unpaid care provided by family caregivers were based on the average salary per hour in each country. More detailed information on the collection of unit cost data is provided in the S2 Section in S1 File.

### Missing data

Once we computed QALYs and total costs, approximately 8% of patients had a missing value for at least one cost category (164 patients) and 26% had at least one missing element required for QALY estimation (523 patients). More details can be found in the S3 Section in S1 File.

We assumed a missing at random (MAR) pattern of missing data. Variables were multiple imputed using multilevel joint modelling, for any missing EQ-5D-5L score and for each cost category rather than for aggregated measures of QALYs and total costs [48]. Patients’ personal characteristics with no (or very few) missing values were used as the basis for imputation, namely: age, sex, education, smoking status, quantity of alcohol consumed, number of drugs at baseline, number of comorbidities at baseline, number of hospitalizations during twelve months prior to baseline, being housebound at baseline, living in a nursing home at baseline, having dementia at baseline, duration of index hospitalization, whether the index hospitalization was in a medical or surgical ward, country, duration of patient follow-up, and whether or not the patient died during the trial. Multiple imputations (MI) were performed separately by treatment arm. Each imputation created five multiple imputed databases, each generated from 100 iterations [49]. We specified an MI model with random intercepts and slopes. Multiple imputed results were estimated according to the combination rules by Rubin [50].

We challenged the MAR assumption by running the main model under several scenarios where a MNAR structure of missing data was assumed [51]. Overall, results were robust and not sensitive to the MAR assumption (see S4 Section in S1 File).

### Robustness checks

Several robustness checks were performed to test and confirm the validity of the main model. Firstly, we computed the simple difference between intervention and comparator arm costs and QALYs without any regression analysis. Secondly, we compared the main GSEM model with a simpler Seemingly Unrelated Regression (SUR) model [52] as well as separate linear mixed models of costs and QALYs. We, then, added interaction terms between arm and country to the main model and performed a likelihood ratio test to compare the two models, with and without the interaction terms.

In a further robustness check, we relaxed the normality assumption of the GSEM model and assumed gamma distributed errors with a log link function. Finally, we ran a CEA including complete observations only, excluding participants for whom incomplete cost or incomplete EQ-5D-5L responses were obtained.

### Country-level and subgroup analyses

We performed country-specific analyses, by applying local costs (converted to CHF) and locally relevant EQ-5D-5L valuation algorithms (as specified above) for each country in turn.

In addition, we performed subgroup analyses for the following subgroups defined in the OPERAM trial protocol [8]: patient’s sex (female versus male), degree of independence (community-dwelling versus living in a nursing home at baseline), medical specialty of clusters (hospitalization in a medical versus surgical ward), age (70–79 years; 80–89 years; more than 90 years), number of drugs (5–9; ≥ 10), number of chronic comorbidities (3–6; ≥7).

### Sensitivity analysis

We reduced and increased the unit costs for each cost category (medical visits, drugs, etc.), in turn, by 30% to assess the impact of related uncertainty on incremental cost-effectiveness. Finally, we combined a non-parametric bootstrap-based estimation of uncertainty ranges and probabilistic sensitivity analysis, by drawing a vector of values from normal distributions representing the parameter uncertainties of the cost parameters, alongside 1’000 bootstrap replications [53]. In each replication, costs were multiplied with the draws resulting from the applicable normal distributions, the main GSEM model was re-estimated and incremental cost and QALY results derived. We drew 1’000 bootstrap samples from each of the 5 imputed datasets separately, then pooled the samples together and presented them in a cost-effectiveness plane [34].

### Technical implementation

All analyses were run in STATA, version 15, apart from the multilevel joint modelling multiple imputation, which was conducted on R software by using the R package JOMO, suitable for cluster-specific covariance matrices (https://www.rdocumentation.org/packages/jomo, [54]).

### Ethical approval

The OPERAM trial/project was approved by the independent research ethics committees at each centre (lead ethics committee: Cantonal Ethics Committee Bern, Switzerland, ID 2016–01200; Medical Research Ethics Committee Utrecht, Netherlands, ID 15-522/D; Comité d’Ethique Hospitalo-Facultaire Saint-Luc-UCL: 2016/20JUL/347–Belgian registration No: B403201629175; Cork University Teaching Hospitals Clinical Ethics Committee, Cork, Republic of Ireland; ID ECM 4 (o) 07/02/17), and by Swissmedic as the responsible regulatory authority.

## Results

Between December 2016 and October 2018, 2,008 participants were allocated to 54 clusters (963 participants) in the intervention group, and 56 clusters (1,045 participants) in the control group. Participants were recruited in Switzerland (822), Belgium (388), Ireland (346) and the Netherlands (452). The median age was 79 years and 898 participants (44.7%) were women. Ten (0.5%) participants were lost to follow-up, 118 (5.9%) participants withdrew from the trial, and 384 (19.1%) died during follow-up.

### Descriptive statistics of costs and QALYs

Descriptive statistics presented in Tables 1 and 2 are based on the observed sample (i.e. non-imputed). Table 1 shows the descriptive statistics for QALYs, estimated using the Germany EQ-5D-5L crosswalk valuation algorithm [25], for the one-year trial observation period for all countries and by country. During the trial observation period, patients in the intervention group and control groups accrued a mean of 0.649 and 0.632 QALYs, respectively. Mean QALYs were higher for intervention patients in Switzerland and Belgium, but lower in Ireland and the Netherlands. In contrast, median QALYs were higher for intervention patients in Switzerland, Ireland and Belgium, but lower in the Netherlands.

**Table 1:** QALYs for all countries and by country per patient over one year.

QALYs	N	Mean	Std. Dev.	Min	Max	Median
***All countries***						
Control arm	765	0.632	0.307	-0.01	1	0.742
Intervention arm	720	0.649	0.312	-0.01	1	0.771
***Switzerland***						
Control arm	285	0.666	0.291	-0.01	1	0.772
Intervention arm	353	0.670	0.316	-0.01	1	0.799
***Ireland***						
Control arm	171	0.650	0.306	0.00	1	0.747
Intervention arm	110	0.625	0.348	0.00	1	0.796
***Belgium***						
Control arm	162	0.597	0.306	0.00	1	0.700
Intervention arm	120	0.697	0.220	0.05	1	0.766
***The Netherlands***						
Control arm	147	0.583	0.334	-0.01	1	0.716
Intervention arm	137	0.574	0.330	0.00	1	0.686

Note: QALYs were estimated over the one-year trial observation period.

**Table 2:** Total medical costs (CHF) per patient over one year.

Total costs (CHF)	N	Mean	Std. Dev.	Min	Max	Median
***All countries***						
Control arm	954	44’767	51’787	27	314’210	24’630
Intervention arm	890	44’353	50’812	94	412’074	23’976
***Switzerland***						
Control arm	345	52’904	54’674	49	314’210	34’721
Intervention arm	427	44’513	48’513	94	313’687	25’693
***Ireland***						
Control arm	202	44’872	51’871	104	257’562	23’865
Intervention arm	125	43’251	55’539	161	412’074	22’148
***Belgium***						
Control arm	204	33’181	42’351	30	307’074	16’927
Intervention arm	136	26’518	37’052	192	253’061	12’530
***Netherlands***						
Control arm	203	42’474	53’181	27	264’802	18’721
Intervention arm	202	56’703	56’892	125	256’195	34’729

Note: local costs expressed in Swiss Francs (CHF) through the PPP index.

Table 2 shows non-adjusted total direct medical costs per patient, which were slightly lower for intervention patients (CHF 44’353) than for control patients (CHF 44’767). In Switzerland, Ireland and Belgium, results showed the same trend of lower direct medical costs for intervention patients, while in the Netherlands medical costs were lower for control patients.

**Fig 1:**
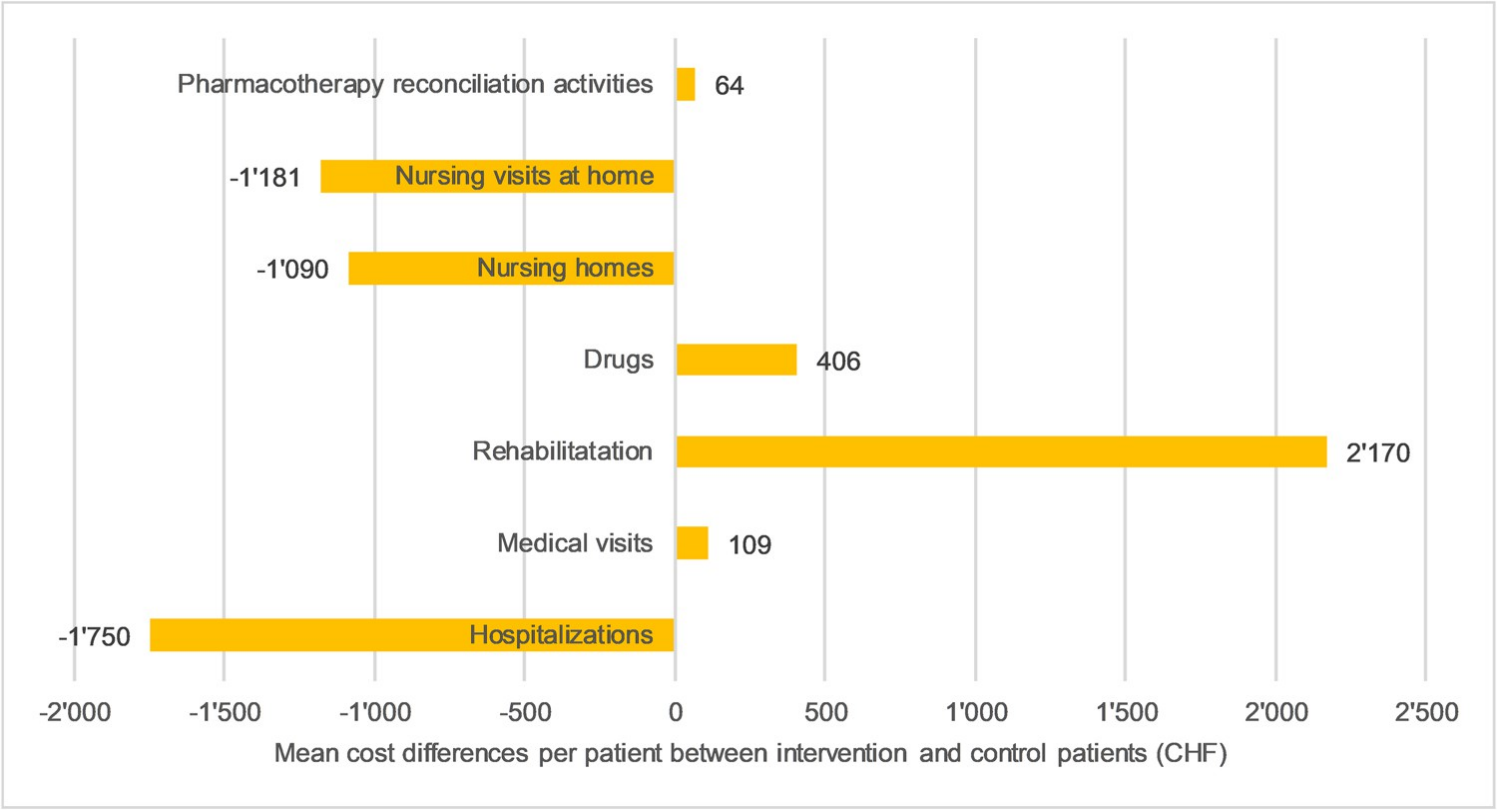
Mean cost differences per patient (CHF) between intervention and control patients, broken down by cost categories, all countries. Note: A positive cost difference (bar on the right hand-side) means that costs were higher in the intervention arm, while a negative cost difference (on the left hand-side), indicates that costs were higher in the control arm.

Fig 1 presents differences by cost category between the trial arms, for all countries combined. Intervention patients were on average more costly in terms of drugs (CHF +406), rehabilitation (CHF +2’170) and medical visits (CHF +109) but less costly in terms of hospitalizations (CHF -1’750), nursing visits at home (CHF -1’181), and stays in nursing homes (CHF -1’090). In the S1 File, further details of the estimated costs are provided (S2-S9 Tables in S1 File).

### Results of cost-effectiveness analyses and country-heterogeneity test

In the main, trial-wide GSEM analysis (Table 3), the STRIP intervention was estimated to generate 0.025 incremental QALYs (95% confidence interval (95% CI): -0.002; 0.052, p-value: 0.068) and reduce health care costs by CHF 3’588 (95% CI: -7’716; 540, p-value: 0.088) (approximately EUR 3’000). Both the qualitative and quantitative heterogeneity tests indicated the presence of between-country heterogeneity for incremental costs, and the qualitative test also for incremental QALY estimates

**Table 3:** Results of the main cost-effectiveness analysis, GSEM models.

	Healthcare system perspective	Societal perspective
**Effects on costs (CHF)**		
Intervention arm	-3’588	-4’214
	[-7’716,540]	[-8’476,48]
Age	800***	946***
	[445,1’156]	[573,1’319]
Female	-34	2’258
	[-4’159,4’090]	[-2’050,6’566]
Utility 6 months before	-4’008	-6’650
	[-17’712,9’696]	[-19’862,6’561]
Utility baseline	-21’033***	-21’897***
	[-30’854,-11’212]	[-31’496,-12’298]
Number of drugs	1’071***	1’168***
	[562,1’581]	[636,1’699]
Number of comorbidities	593**	601**
	[215,971]	[210,991]
Housebound	3’218	3’402
	[-3’430,9’867]	[-3’195,10’000]
Smoker	1’062	3’148
	[-6’654,8’779]	[-4’807,11’104]
High School	1’303	-235
	[-3’760,6’366]	[-5’452,4’982]
University	1’397	269
	[-4’600,7’394]	[-5’857,6’396]
Living in nursing home	49’600***	46’151***
	[40’042,59’157]	[36’425,55’878]
Dementia	3’462	516
	[-6’246,13’170]	[-9’417,10’450]
N. of hosp. 1 year before	2’861***	2’784***
	[1’427,4’296]	[1’309,4’259]
Medical ward	7’938**	9’003**
	[2’426,13’449]	[3’202,14’804]
Observation time	141***	156***
	[124,159]	[137,175]
Duration baseline hosp.	489***	517***
	[309,669]	[333,702]
Ireland	-299	11’507**
	[-7’085,6’487]	[4’312,18’702]
Belgium	-7’789*	-7’492
	[-14’977,-600]	[-15’027,43]
Netherlands	9’293**	11’793***
	[2’863,15’723]	[4’947,18’638]
Constant	-85’427***	-100’042***
	[-116’461,-54’392]	[-132’669,-67’414]
**Effects on *QALYs***		
Intervention Arm	0.025	0.025
	[-0.001,0.052]	[-0.001,0.052]
Age	-0.006***	-0.006***
	[-0.008,-0.004]	[-0.008,-0.004]
Female	-0.000	-0.000
	[-0.035,0.035]	[-0.035,0.035]
Utility 6 months before	0.191***	0.191***
	[0.131,0.252]	[0.131,0.252]
Utility baseline	0.316***	0.316***
	[0.265,0.367]	[0.265,0.367]
Number of drugs	-0.007***	-0.007***
	[-0.010,-0.004]	[-0.010,-0.004]
Number of comorbidities	-0.003*	-0.003*
	[-0.005,-0.000]	[-0.005,-0.000]
Housebound	-0.056*	-0.056*
	[-0.099,-0.012]	[-0.099,-0.012]
Smoker	0.016	0.016
	[-0.032,0.066]	[-0.032,0.066]
High School	0.004	0.004
	[-0.033,0.042]	[-0.033,0.042]
University	0.028	0.028
	[-0.015,0.071]	[-0.015,0.071]
Living in nursing home	0.013	0.013
	[-0.068,0.095]	[-0.068,0.095]
Dementia	-0.005	-0.005
	[-0.082,0.070]	[-0.081,0.071]
N. of hosp. 1 year before	-0.013**	-0.013**
	[-0.022,-0.004]	[-0.022,-0.004]
Medical ward	-0.065**	-0.066**
	[-0.105,-0.026]	[-0.105,-0.026]
Duration baseline hosp.	-0.003***	-0.003***
	[-0.005,-0.002]	[-0.005,-0.002]
Ireland	-0.029	-0.029
	[-0.085,0.027]	[-0.085,0.026]
Belgium	-0.036	-0.036
	[-0.086,0.013]	[-0.086,0.013]
Netherlands	-0.051*	-0.051*
	[-0.094,-0.008]	[-0.094,-0.008]
Constant	1.037***	1.039***
	[0.844,1.231]	[0.845,1.232]
Observations	2008	2008

Note: GSEM models. 95% confidence intervals in brackets.

* p<0.05

** p<0.01

*** <0.001. Local costs are expressed in Swiss Francs (CHF) and combined using purchasing power parity indices (PPP). The main results of the GSEM-based analysis, i.e. the incremental costs and incremental QALYs representing differences between the intervention and control arms, are equivalent to the coefficients of the variable “intervention arm”. Results always represent average values per patient. A positive value of the coefficient for `intervention arm`, for costs/QALYs, indicates that the intervention is associated with higher average costs/QALYs per patient, and vice versa. Intervention arm, female, housebound, smoker, nursing home (i.e. living in a nursing home at baseline), dementia and medical ward (whether the baseline hospitalization occurred in a medical vs surgical ward) are dichotomous variables. Switzerland, Ireland, Belgium and the Netherlands form parts of a categorical variable; Switzerland serves as the reference group. Education (less than high school, high school, university) is a categorical variable; less than high school serves as the reference group. Age is measured in years; utility 6 months before baseline and utility at baseline are ranged from -0.2 to 1; number of drugs (at baseline), number of comorbidities (at baseline) and number of hospitalizations (in the year prior to baseline) are integers; observation times and duration of baseline hospitalization (duration baseline hosp.) are measured in days.

Table 3 shows the results of the main, GSEM-based CEA of the overall trial, including covariate effects. Numerically, the intervention strategy was dominant over the control strategy but the intervention arm effects on QALYs and costs were not statistically significant. Adoption of the approximated societal perspective in which informal care costs were additionally included, resulted in incremental costs of CHF -4’214 (95% CI: -8’476; 48), in favour of the intervention strategy, compared to CHF -3’588 (95% CI: -7’716; 540) for the healthcare system perspective. The directions of associations of covariates with total healthcare costs and total QALYs were all as expected. All results shown are multiple imputation-based, except where stated otherwise.

### Country-specific and subgroup analyses

Table 4 shows the incremental costs and incremental QALYs (i.e. the coefficients for intervention arm) obtained in the country-specific and subgroup analyses. Full results are provided in the S10-S16 Tables in S1 File.

**Table 4:** Results of cost-effectiveness analyses for countries and subgroups, coefficients of main interest only, local costs (expressed in CHF).

	Incremental Costs	95% CI	Incremental QALYs	95% CI	ICER
*Analyses by country*					
Switzerland (CHF)	-7’027*	[-13’130–924]	0.068	[-0.038 0.052]	Dominant
Ireland (CHF)	-8’963	[-20’373 24’456]	-0.006	[-0.072 0.059]	1’493’833
Belgium (CHF)	-6’081	[-17’073 4’910]	0.023	[-0.064 0 .111]	Dominant
The Netherlands (CHF)	5’758	[5’273 16’789]	0.074	[-0.002 0.151]	77’810
*Subgroup analyses*					
Only female	-3’642	[-9’983 2’699]	0.003	[-0.040 0.045]	Dominant
Only male	-4’270	[-9’763 1’222]	0.045	[0.006 0.083]	Dominant
Community-dwelling	-3’081	[-7’344 1’182]	0.024	[-0.002 0.048]	Dominant
Nursing homes	-902	[-15’013 13’207]	0.069	[-0.052 0.189]	Dominant
Medical ward	-4’615	[-9’719 488]	0.019	[-0.010 0.049]	Dominant
Surgical ward	-1’046	[-10’463 8’370]	0.044	[-0.021 0.108]	Dominant
Age 70–79	-2’547	[-7’740 2’646]	-0.001	[-0.041 0.039]	2’791’232
Age 80–89	-5’293	[-12’497 1’909]	0.063	[0.021 0.105]	Dominant
Age 90+	984	[-13’668 15’637]	0.047	[-0.078 0.173]	20’793
N. drugs: 5–9	-3’884	[-10’667 2’899]	0.009	[-0.035 0.053]	Dominant
N. drugs: ≥ 10	-3’177	[-8’548 2’192]	0.038	[0.002 0.073]	Dominant
N. comorbidities: 3–6	4’388	[-6’349 15’127]	-0.029	[-0.105 0.046]	Dominated
N. comorbidities: ≥ 7	-5’964	[-10’811–1’117]	0.034	[0.005 0.063]	Dominant

Note: the first column characterizes the analysis performed. All analyses were run with the same set of covariates as were used for the main analysis. Column 2 and column 4 report the coefficients of the variable “Intervention arm”, representing incremental costs and incremental QALYs respectively. ICER: incremental cost-effectiveness ratio in CHF per QALY gained. The positive ICER value relative to the analyses for “Ireland” and “Age group 70–79” represent saving per QALY lost.

The country-specific analyses yielded very similar incremental costs for Switzerland (CHF -7’026, 822 patients), Ireland (CHF -8’963 equal to EUR -8’150, 346 patients) and Belgium (CHF -6’081 equal to EUR -5’530, 388 patients), but very different ones for the Netherlands (CHF 5’758 equal to EUR 5’234, 452 patients).

Overall, the results of the subgroup analyses were consistent with the main results (Table 4). They may suggest that the potential cost savings and increase in QALYs in the intervention arm, were higher for patients with a more serious clinical situation. For instance, having at least seven comorbidities corresponded with an estimate of mean incremental costs of CHF -5’964 (95% CI: -10’811; -1’117), and patients taking at least ten drugs were estimated to generate mean incremental QALYs of 0.038 (95% CI: 0.002; 0.073). The intervention was also estimated to generate a statistically significant increase in QALYs for the male subgroup (0.045, 95% CI: 0.006; 0.083), and for the subgroup aged 80–89 years (0.063, 95% CI: 0.021; 0.105).

### Robustness checks

Results of additional robustness checks for the main analysis are shown in the S1 File. Compared to the GSEM-based analysis, the simple, non-multivariable-adjusted CEA presented smaller incremental costs (CHF -1’486, 95% CI: -6’153; 3’180) and similar incremental QALYs (0.026, 95% CI: -0.005; 0.057, S17 Table in S1 File).

The SUR model (incremental costs: CHF -3’822, 95% CI: -7’970; 326, and incremental QALYs: 0.025, 95% CI: -0.001; 0.052, S19 Table in S1 File, column 1) and separate linear mixed models of costs and QALYs (CHF -3’670, 95% CI: -7’662; 321, and incremental QALYs: 0.025, 95% CI: -0.002; 0.052, S19 Table in S1 File, column 2) yielded results similar to those of the main GSEM model. In a further robustness check, we added an interaction term between arm and country to the main model, which was not statistically significant (details not shown). The model with gamma distributed errors with a log link function resulted in mean incremental costs and QALYs similar to the main analysis (S18 Table in S1 File). Results obtained with the non-imputed dataset (only observed data included) resulted in incremental costs of CHF -4’022 (95% CI: -8404; 360) and incremental QALYs of 0.017 (95% CI: -0.007; 0,044), which were close to the results from the main analysis (S19 Table in S1 File, column 3).

### Sensitivity analyses

**Fig 2:**
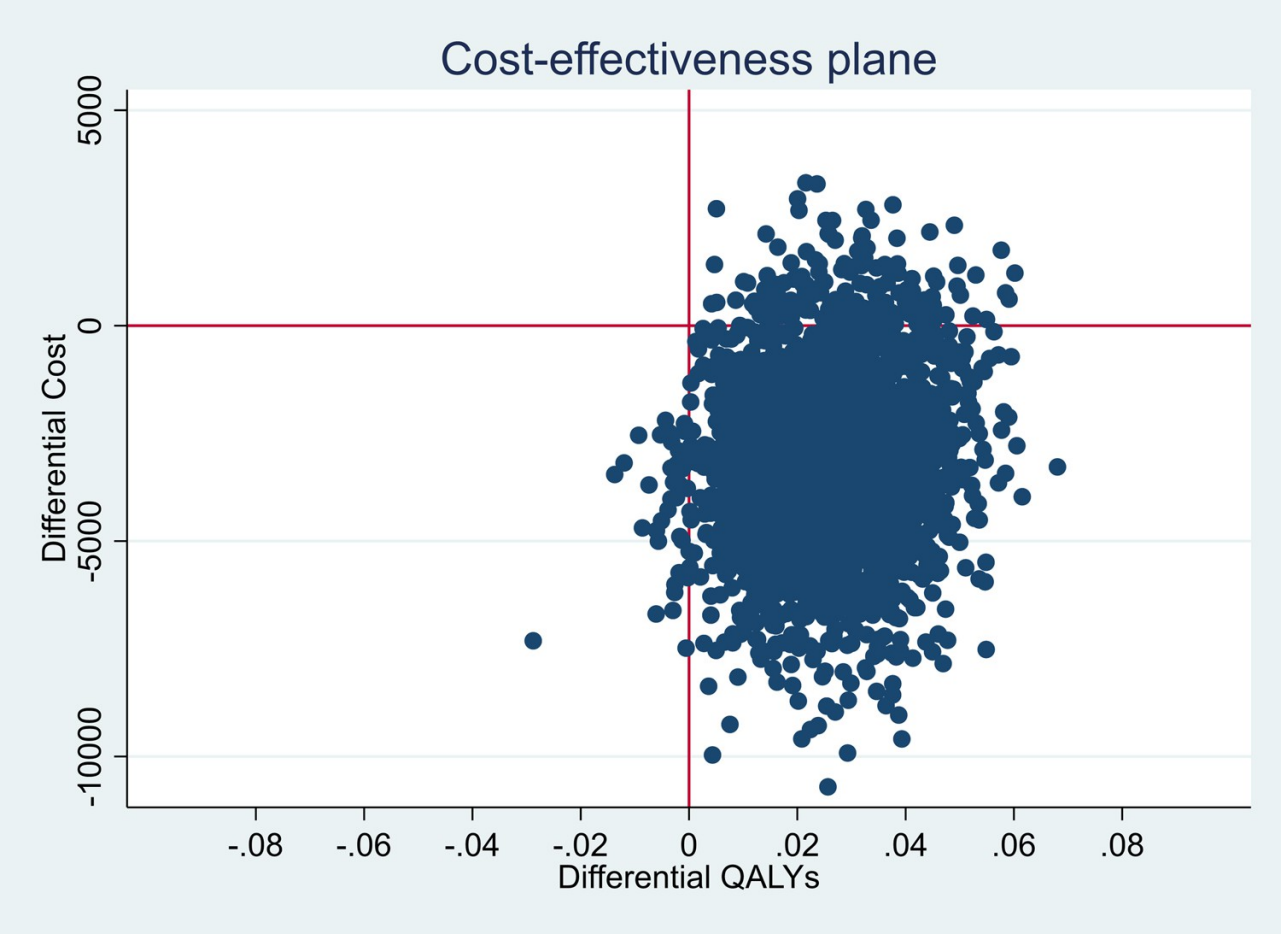
Cost-effectiveness plane for all countries. Note: The X-axis shows the difference in QALYs between the OPERAM trial arms: a positive QALY difference means an increase in QALYs in the intervention arm and is represented on the right-hand side. By contrast, a negative QALY difference is represented on the left-hand side. The Y-axis shows the cost differential. If the intervention is associated with a cost reduction, the cost differential is negative (lower part of the graph), while a positive cost differential (upper part of the graph) indicates an increase in costs due to the intervention.

Deterministic sensitivity analysis of unit cost parameters, showed no substantial effect from varying these parameters (S20 Table in S1 File). Fig 2 shows the results of the combined bootstrap and probabilistic sensitivity analysis. The majority of the bootstrap replications (92.4%) were in the lower right quadrant of the cost-effectiveness plane, indicating dominance of the intervention.

## Discussion and conclusion

We assessed the cost-effectiveness of the OPERAM randomized clinical trial intervention STRIP to reduce potentially inappropriate prescribing and DRAs, using a software-based pharmacotherapy optimization intervention based on the STOPP/START criteria [14]. By doing this, we have built on a recent strand of literature dealing with the optimization of pharmacotherapy as a means of reducing hospital admissions and improving other patient-relevant outcomes. Based on the health economic data collected in the OPERAM trial, the focus of our analysis was on the Swiss healthcare system, with complementary analyses undertaken for the other countries participating in OPERAM.

The OPERAM trial intervention was numerically dominant, as it resulted in cost savings of about CHF 3’500 per patient and a gain of about 0.025 QALYs per patient over the one-year trial observation period. However, these estimates of incremental costs and QALYs were not statistically significant. Results of the analysis from a societal perspective suggested an even higher potential cost saving per patient (CHF -4’214). Of note, the coefficient for Ireland differed substantially between the two analyses (CHF -299 vs CHF 11’507 when adding the opportunity cost of informal care) due to a strong reliance on informal care in the country (see also S8 Table in S1 File). Positive results of our analysis do not contradict the main outcome of the OPERAM trial [10]. Although the trial did not find a statistically significant reduction in DRAs resulting from the STRIP intervention, there was some evidence in terms of successfully implemented, pharmacotherapy-related recommendations in 474 (51.7%) participants in the intervention group. Also, the hazard ratios for specific secondary outcomes of the trial were not statistically significant but quite consistently in the direction of favouring the intervention (0.96 for the first fall; 0.90 for death). We speculate that the combination of these non-significant but numerically positive results, suggesting intervention effectiveness, may have translated into our estimates of higher QALYs and lower health care utilisation costs.

Previous studies addressing effectiveness of medication-optimising interventions mainly focused on medical outcomes and yielded very mixed evidence. Our results for costs can be compared with two Cochrane reviews looking at interventions to optimise prescribing for older adults in nursing homes that also looked at the effects on drug costs [5]. In one of the reviews, 12 relevant studies were identified of which only 5 found that medication optimising interventions were associated with a reduction in drug costs (and this reduction was small in all 5 studies) [5]. In the other review which assessed pharmacist-directed medication management interventions for older adults in nursing homes, non-significant cost reductions were found to result from intervention [6], which is similar to the results of this study.

Results by country were heterogeneous. They went in the same direction for Switzerland, Belgium and Ireland where intervention arm costs were lower. However, in the case of the Netherlands, the intervention arm showed higher costs. We could not find a substance-matter explanation for this deviation from the overall trend. It could to some extent be due to differences between countries in the way primary care physicians received and implemented the STRIPA-based recommendations, generated in the inpatient setting. It is also possible that some primary care physicians were more compliant, others less so, with STRIPA recommendations. Observed heterogeneity between centres/countries, which may have resulted from health system characteristics and resulting subtle differences in the implementation of the intervention, represents a limitation of the trial. Therefore, the results of our CEA should be interpreted with caution and consider local implementation. Subgroup analyses suggested more favourable results for groups of persons at higher risk (i.e. having at least seven comorbidities or taking at least ten drugs). It should be noted, however, that patient numbers for the country-level and subgroup analysis were quite limited, and we cannot exclude an increased probability of chance effects.

We undertook a fully pre-planned health economic data collection and approach to analysis. The methodological approaches pursued may inform the conduct of other CEAs of cluster-randomized trials with similar design and validity issues. In our multiple imputation of missing data, we explicitly accounted for the clustered nature of the data. We subsequently used a regression-based approach (GSEM) to take into account the simultaneity and thus, potential correlation, of costs and QALY results for each patient, and also the presence of clusters given trial design. Robustness checks confirmed the validity of the GSEM approach.

We also detected between-country heterogeneity in the main outcomes. We included country fixed effects in the main GSEM analysis, where we were primarily interested in the overall effect of the treatment. In addition, we ran separate CEAs for each country to further address the heterogeneity issue (see S10 Table in S1 File). Other analysis methods reported in the literature recommend adding interaction terms between arm and country (as we did in a robustness check), or consider countries as random effects [13]. We omitted the latter option given the relatively small country-level sample sizes and small number of countries in the OPERAM trial. Also, it is common in CEAs of multinational trials to apply the unit costs of one country to all participating countries [20, 55, 56]. We alternatively applied local costs to each country and made them comparable through the use of PPP. This approach has been reported to better capture differences in mean, spread and skewness across centres [13, 31–33].

This study had some limitations. Firstly, the presence of missing data, despite being addressed through the multiple imputation procedure, remains a potential source of bias for our analyses. Secondly, there were some unclear data points in the trial database, especially in the drug utilisation data. In about a quarter of cases, values representing dose, unit of dose, or start or end date information were unclear and had to be treated as missing. This may have led to data distortion to some extent. Thirdly, giving the absence of a Swiss EQ-5D valuation algorithm, the main analysis used a German algorithm on the basis of geographic proximity [57]. Finally, the sample size of the trial was based on the clinical primary endpoint, i.e. drug-related hospital admissions, and may have been insufficient to detect statistically significant changes in economic outcomes, particularly QALYs.

There is a recognized lack of research on the economic impact of pharmacotherapy optimisation, and inconsistency in the little available evidence on the effects of related interventions for both costs and effectiveness. This study emphasizes the relevance of including CEA in clinical trials and other studies aimed at optimizing pharmacotherapy. The OPERAM trial intervention was numerically dominant, with a potential saving of about CHF 3’500 and a gain of 0.025 QALYs per patient over a one-year time horizon. However, results were not statistically significant at the 5% level. Hence, more research is needed to support policy makers to address resources in an efficient way. The methodological approaches of the present CEA could be used to inform the planning of future CEAs of multi-country, cluster-randomized trials facing similar challenges.

## Supporting information

S1 File (DOCX)
