# Supplementary material for: Cost-effectiveness of a structured medication review approach for multimorbid older adults: Within-trial analysis of the OPERAM study
Source: PLoS One. 2022 Apr 11;17(4):e0265507. doi: 10.1371/journal.pone.0265507 (PMC9000111; doi:10.1371/journal.pone.0265507)
Supplement: S1 File — (DOCX) [file pone.0265507.s001.docx]

Supplementary materials

S1 DEVIATIONS FROM THE PRE-PLANNED APPROACH

Deviations from the pre-planned Health Economics Analysis Plan (HEAP) were very few. Given dominant results, we did not compute uncertainty ranges (confidence intervals) for incremental cost-effectiveness ratios (ICERs). Given the not clear-cut results, we performed some additional analyses not pre-specified in the HEAP, and we slightly changed the methodological approach to additional checks. Among the descriptive and non-adjusted analyses on costs, we did not test for univariate differences between trial arms based on non-parametric bootstrapping, given sufficient coverage of this aspect in the regression analyses. The HEAP also specified that sensitivity analyses might be added to challenge the MAR assumption. Since data were homogeneously distributed across arms, and the occurrence of missing data was at a quite expected level (25% of observations), we decided to omit this part.

S2 collection of Unit cost data

Unit costs were drawn from external sources, i.e. not collected directly in the OPERAM trial. All costs for all countries refer to year 2018.

The collection of Swiss unit cost data was performed as follows:

Hospitalizations were estimated using diagnosis-related group-based reimbursement (1). Two approaches were pursued and compared:

In order to estimate a Swiss unit cost for all hospitalizations in OPERAM (irrespective of country), ICD-10 diagnosis codes from the OPERAM trial database were linked to Swiss diagnosis-related group codes. Such diagnosis codes were directly available for OPERAM follow-up hospitalizations. The link between ICD and DRG codes was performed based on a large set of hospitalizations at University Hospital Bern. For validation and comparison, we additionally used a second dataset provided by Canton Basel-Stadt, Switzerland. All these datasets were anonymous.

In a second approach, the diagnosis-related group codes assigned to OPERAM hospitalizations at University Hospital Bern were directly requested.

Costs of outpatient physician visits by specialty, and for visits with other healthcare providers (e.g. physiotherapists), were provided by a large provider of Swiss statutory health insurance.

Costs of nursing visits at home, nursing home care and rehabilitation facilities were drawn from national statistical data. In particular, costs of nursing home care and rehabilitation facilities were based on statistics available from the Swiss National Statistical Office (2). Data on nursing visits at home were obtained through additional contacts (calls and e-mail exchanges) with an officer of the Federal Office of Public Health who provided extra material useful to derive a sufficiently accurate cost estimate.

Drug costs were drawn from official data sources (mainly (3)). For each ATC code, a cost per unit of measure was computed and derived as an average cost based on all different versions of each drug corresponding to the respective ATC code, accounting for different brands/producers, different units of measure, different doses of the active substance contained.

The costing of the STRIP intervention was based on the estimated cost of the software and staff costs.

To estimate the cost of the software per patient, we first performed a review of similar tools already available on the market. We identified eight software tools with similar aims as STRIPA. For three of them, it was possible to find pricing information online. Based on the three prices, we computed a marginal cost per patient equal to zero. However, we decided to assume a cost of CHF 5 per patient, to be conservative.

Staff costs per unit of time spent for activities related to reconciliation of medication were provided by each country. The data collection considered the time spent on the following activities, for the patients of both trial arms: recording of medication at admission, reconciliation of medication. For treated patients the time for the following activities was added: drug review, discussion with the prescribing physician, shared decision making, revision of the STRIPA results during the hospital stay. Due to a data collection issue occurring during the process, data on time recording were not only collected for trial patients but also for other patients with similar characteristics.

The collection of Irish unit cost data by UCC with support from UNIBAS was performed as follows:

Hospitalization costs were estimated using disease-related group-based reimbursement. The Irish Health Service Executive Ready Reckoner Casemix report (4) was used to assign a cost to each ICD-10 diagnosis code recorded in the OPERAM database.

Costs of outpatient visits by specialty and other outpatient services were drawn directly from studies supplied by the hospital finance department (5).

Costs of visits to general practitioners were based on the standard fee in Ireland for a general practitioner visit.

Costs of nursing visits at home, nursing home stay and rehabilitation stays were drawn from Health Service Executive data (6).

Drug costs were estimated using a pre-purchased wholesaler price list.

Costs of STRIP intervention was calculated based on time estimates and Health Service Executive public pay scales (7).

The collection of Belgian unit cost data was performed as follows:

Costing was performed as much as possible in line with the national guideline for health-economic research (8).

- Unit costs for hospitalizations were retrieved from coupled and validated data from the Minimal Hospital Data of the Public Federal Service Public Health and invoicing data of the National Institute for Health and Disability Insurance (NIHDI) for year 2017 (national database medical diagnosis/care and cost (9). Only the cost of hospitalizations for patients over 65 years were included.
- Costs of specialist visits, GP visits and other outpatient services were taken from the NIHDI website (10).
- Approaches to costs of nursing visits at home, nursing home stays and rehabilitation stays were taken from national statistical data (10).
- Costs of informal, unpaid care were based on the average salary per hour in Belgium retrieved from the Belgian national office of statistics (11).
- Drug costs were estimated using the costs available on the website of the “Centre Belge d’Information Pharmacothérapeutique (CBIP)” in 2018 (12). The costs were computed for each ATC code based on the route of administration of the drug and unit of measure.

The collection of Dutch unit cost data was performed as follows:

Costing was performed as much as possible in line with the national guideline for health-economic research (13).

- Hospitalization costs were estimated using the Dutch manual for costing studies in healthcare. Data referred to 2014 but were corrected for inflation to represent 2018 values (14).
- Costs of outpatient visits by specialty, of visits to general practitioners and other outpatient services were based on national costing studies (14).
- Costs of nursing visits at home, nursing home stays and rehabilitation stays were based on national costing studies for relevant care units (14).
- Drug costs were adapted from the Belgian drugs costs to the Netherlands, using a conversion scale of costs specific to pharmaceutical products elaborated by the European Parliament (15).
- Costs of STRIP intervention were calculated based on time estimates and Health Service Executive public pay-scales (16).

S3 APPROACH TO missing data

Missing data, particularly relating to quality of life and medical resource use, are very often an issue in trial-based cost-effectiveness analyses. The scientific literature does not recommend the adoption of ad-hoc solutions to solve missing data problems, but rather to perform statistical multiple imputation on the whole database. Before performing the statistical multiple imputation, it was necessary to define and apply some rules to the overall database. This was done as follows.

- For some categories of costs (relating to hospitalizations, rehabilitation facilities, medical visits, nurses’ visits at home), the information on number and duration of visits was collected during the three OPERAM trial follow-up visits. Therefore, for each of these categories, we first computed the cost for each of the three time periods by multiplying the cost per visit and the number of visits (or length of stay) in the relevant period. We then obtained the total cost of each category by summing up the three partial costs for each follow-up period. In some cases, the partial costs for one or two of the three time periods were missing. We considered these cases as zeros. The percentage of such missing values were always in the range of 10-15% of total observations; occurrence was always equally distributed across arms. Only if all the three components were treated as missing, the whole cost per patient was missing and thus multiple imputed. This decision was made to avoid imputation of a huge number of variables with the correlated risk of losing precision and of substantially slowing down the imputation process.
- For nursing home stays, we assumed that if two out of three answers regarding length of stay were zero and one missing, they were all zero.
- For drug costs, we applied a similar approach as for the other cost categories. In case of implausible values of dose or unit of measure, a missing value was assigned. In case of missing drug dose, start date or end date information, or unit of measure, the information on the drug cost was treated as missing. Drug data entries with at least one missing piece of information amounted to 23% of the total, equally distributed across arms. Not all, but most of the missing information was for inexpensive drugs such as nutrients, creams, disinfectants, food supplements, etc. The cost of each drug was summed up for each patient to obtain a total drug cost per patient. Similar as in the case of the medical cost, missing drug costs were treated as zeros when summed up at patient-level. Thus, in the end, the drug cost per patient was only missing for patients who had missing values for all drugs taken. This occurred in 3% of the patients.
- For hospitalizations occurred after the baseline, the ICD-10 diagnosis codes from the OPERAM trial database were linked to Swiss diagnosis-related group codes obtained from University Hospital Bern. For about half of these hospitalizations it was not possible to find an exact correspondence. We also tried a less accurate matching where only the first letter and two numbers of the ICD-10 code needed to match, rather than the whole ICD code. In this way, the number of non-matched records (where the mean was imputed) was dramatically reduced.
- For the EQ-5D-based utilities, we multiple imputed any missing value. An alternative approach often followed in the literature could have been to first compute quality-adjusted life years (QALYs) and total costs, and impute only these two final variables. However, we wanted to use as much information as possible. This is why we opted for a multiple imputation of single EQ-5D-based utilities and cost categories rather than aggregated measures as QALYs and total costs.

After these steps, we had 535 patients (26%) with missing data if values of EQ-5D-based utility and costs were considered together. We assumed a missing at random (MAR) pattern of missing values. We performed a multilevel multiple imputation with the R package JOMO, the only available software to use cluster-specific covariance matrices (<https://www.rdocumentation.org/packages/jomo>). JOMO is based on joint modelling multiple imputation. We specified a model with random intercept and random slope. The package is designed to work on and to provide a Stata-format output (.dta).

We multiple imputed the variables for each cost category and for the utility at baseline, 6 months before baseline, and at each follow-up time point. The patients’ personal characteristics with no (or very few) missing values were used as the basis to statistically impute the variables with missing values. They were: age, sex, education, smoking status, quantity of alcohol consumed, number of drugs at baseline, number of comorbidities at baseline, number of hospitalizations during twelve months prior to baseline, being housebound at baseline, living in a nursing home at baseline, having dementia at baseline, duration of index hospitalization, index hospitalization in a medical (versus surgical) ward, country, follow-up time, death on study.

Multiple imputations were performed separately by arm. Each imputation created five multiple imputed databases, each generated from 100 iterations. We then analysed the database in STATA, version 15. We first created the variable “total cost”, summing up all the cost variables, and the “QALY” variable based on the utility values. Both “gen” commands were preceded by the command “mi xeq:”. We then performed regression-based analyses specifying “xi: mi estimate” before the gsem command, implying consideration of Rubin’s rules (17).

S4 Sensitivity analysis for the MAR assumption of the multiple imputation

We challenged the assumption of missing values being Missing at Random (MAR) and tested a Missing Not at Random (MNAR) assumption. We followed the “*Pattern-mixture models*” approach that tests the hypothesis of different distributions between the missing and observed data (18). In particular, the distribution of a variable is supposed to be a combination of the distribution of the observed and missing values (‘pattern-mixture’). For example, patients with missing data might have a 10% lower quality of life than those observed. To obtain the imputed data under MNAR, we rescaled each MAR-imputed value, i.e. multiplied imputed QALYs and costs by a constant factor, as in Leurent et al (18). We ran 7 scenarios where we changed one of the two parameters or both in parallel. Table 1S shows the results obtained for each scenario.

Table 1S: Sensitivity analysis of missing at random (MAR) assumption

| **Scenario** | **MNAR rescaling parameters** | **Incremental cost (CHF)** | **CI** | **Incremental QALY** | **CI** |
| --- | --- | --- | --- | --- | --- |
|  |  |  |  |  |  |
| 1 | Main analysis | -3602 | [-7791 586] | 0.025 | [-0.003 0.053] |
| 2 | - 10% QALY in both arms | -3583 | [-7763 597] | 0.024 | [-0.003 0.051] |
| 3 | + 10% costs in both arms | -3665 | [-7906 576] | 0.025 | [-0.003 0.053] |
| 4 | - 10% QALY in both arms, + 10% costs in both arms | -3642 | [-7872 589] | 0.024 | [-0.003 0.051] |
| 5 | - 10% QALY in treated | -3639 | [-7826 547] | 0.017 | [-0.010 0.045] |
| 6 | - 10% QALY in controlled | -3547 | [-7727 633] | 0.032 | [0.004 0.059] |
| 7 | + 10% costs in treated | -3589 | [-7802 625] | 0.025 | [-0.003 0.053] |
| 8 | + 10% costs in controlled | -3678 | [-7894 537] | 0.025 | [-0.003 0.053] |

The sensitivity analysis confirmed the consistency of results in case of departures from the MAR assumption. Results were quite stable. Incremental costs ranged from CHF -3547 to -3678 and incremental QALYs from 0.017 to 0.032. Only scenario 6 showed a difference in QALYs results that was statistically significant. Overall, results showed no relevant sensitivity to the MAR assumption.

S5 Additional analyses

*5.1 descriptive statistics*

Descriptive statistics presented in Tables 2S-8S are based on the observed sample (i.e. non-imputed).

Table 2S. Costs of follow-up hospitalizations per patient (CHF)

| **Costs of follow-up hospitalizations (CHF)** | N | Mean | Std. Dev. | Min | Max | Median |
| --- | --- | --- | --- | --- | --- | --- |
|  |  |  |  |  |  |  |
| ***All countries*** |  |  |  |  |  |  |
| Control arm | 1'041 | 14'632 | 25'439 | 0 | 257'527 | 0 |
| Intervention arm | 958 | 12'882 | 24'491 | 0 | 382'154 | 0 |
| ***Switzerland*** |  |  |  |  |  |  |
| Control arm | 372 | 13'041 | 18'204 | 0 | 106'185 | 0 |
| Intervention arm | 441 | 13'389 | 19'467 | 0 | 120'429 | 5'363 |
| ***Ireland*** |  |  |  |  |  |  |
| Control arm | 208 | 20'090 | 32'933 | 0 | 223'901 | 4'486 |
| Intervention arm | 138 | 20'769 | 43'238 | 0 | 382'154 | 0 |
| ***Belgium*** |  |  |  |  |  |  |
| Control arm | 238 | 18'673 | 33'146 | 0 | 257'527 | 0 |
| Intervention arm | 150 | 10'561 | 24'339 | 0 | 187'292 | 0 |
| ***The Netherlands*** |  |  |  |  |  |  |
| Control arm | 223 | 7'884 | 14'085 | 0 | 82'413 | 0 |
| Intervention arm | 229 | 8'673 | 14'707 | 0 | 93'008 | 0 |

Note: Local costs expressed in Swiss Francs (CHF) using purchasing power parities. The min cost is zero because some patients did not have follow-up hospitalizations.

Table 3S. Costs of medical visits per patient (CHF)

| **Costs of medical visits (CHF)** | N | Mean | Std. Dev. | Min | Max | Median |
| --- | --- | --- | --- | --- | --- | --- |
|  |  |  |  |  |  |  |
| ***All countries*** |  |  |  |  |  |  |
| Control arm | 996 | 2'252 | 2'147 | 0 | 12'925 | 1'733 |
| Intervention arm | 921 | 2'360 | 2'243 | 0 | 20'448 | 1'744 |
| ***Switzerland*** |  |  |  |  |  |  |
| Control arm | 366 | 1'976 | 1'895 | 0 | 11'266 | 1'436 |
| Intervention arm | 440 | 1'859 | 1'734 | 0 | 12'132 | 1'429 |
| ***Ireland*** |  |  |  |  |  |  |
| Control arm | 207 | 2'065 | 1'710 | 0 | 11'076 | 1'844 |
| Intervention arm | 133 | 1'899 | 1'665 | 0 | 7'149 | 1'544 |
| ***Belgium*** |  |  |  |  |  |  |
| Control arm | 217 | 2'241 | 2'113 | 0 | 11'714 | 1'643 |
| Intervention arm | 141 | 3'070 | 2'685 | 0 | 20'448 | 2'653 |
| ***The Netherlands*** |  |  |  |  |  |  |
| Control arm | 206 | 2'939 | 2'772 | 0 | 12'925 | 2'080 |
| Intervention arm | 207 | 3'239 | 2'756 | 0 | 15'058 | 2'729 |

Note: Local costs expressed in Swiss Francs (CHF) using purchasing power parities. These costs include costs for specialist visits, primary care physician visits, physiotherapy and emergency room visits.

Table 4S. Costs of rehabilitation facilities per patient (CHF)

| **Costs of rehabilitation facilities (CHF)** | N | Mean | Std. Dev. | Min | Max | Median |
| --- | --- | --- | --- | --- | --- | --- |
|  |  |  |  |  |  |  |
| ***All countries*** |  |  |  |  |  |  |
| Control arm | 993 | 6'223 | 16'498 | 0 | 251'733 | 0 |
| Intervention arm | 920 | 8'393 | 20'920 | 0 | 285'324 | 0 |
| ***Switzerland*** |  |  |  |  |  |  |
| Control arm | 363 | 9'368 | 15'044 | 0 | 103'168 | 0 |
| Intervention arm | 440 | 8'747 | 19'980 | 0 | 285'324 | 0 |
| ***Ireland*** |  |  |  |  |  |  |
| Control arm | 207 | 1’740 | 4'806 | 0 | 32'811 | 0 |
| Intervention arm | 133 | 1'350 | 3'913 | 0 | 28'667 | 0 |
| ***Belgium*** |  |  |  |  |  |  |
| Control arm | 216 | 1'014 | 2'882 | 0 | 28’036 | 0 |
| Intervention arm | 141 | 1'054 | 2'556 | 0 | 17’455 | 0 |
| ***The Netherlands*** |  |  |  |  |  |  |
| Control arm | 207 | 10'626 | 28'208 | 0 | 251'733 | 0 |
| Intervention arm | 206 | 17'208 | 30'744 | 0 | 173'324 | 0 |

Note: Local costs expressed in Swiss Francs (CHF) using purchasing power parities.

Table 5S. Costs of drugs per patient (CHF)

| **Costs of drugs (CHF)** | N | Mean | Std. Dev. | Min | Max | Median |
| --- | --- | --- | --- | --- | --- | --- |
|  |  |  |  |  |  |  |
| ***All countries*** |  |  |  |  |  |  |
| Control arm | 1'009 | 5'474 | 15'083 | 0 | 253'572 | 2'622 |
| Intervention arm | 934 | 5'881 | 16'079 | 0 | 184'219 | 2'491 |
| ***Switzerland*** |  |  |  |  |  |  |
| Control arm | 371 | 5'486 | 21'586 | 1 | 253'572 | 1'912 |
| Intervention arm | 445 | 3'903 | 12'005 | 2 | 150'768 | 1'782 |
| ***Ireland*** |  |  |  |  |  |  |
| Control arm | 207 | 6'969 | 13'064 | 10 | 159'576 | 4'098 |
| Intervention arm | 131 | 6'261 | 10'098 | 1 | 79'464 | 4'218 |
| ***Belgium*** |  |  |  |  |  |  |
| Control arm | 221 | 4'706 | 7'302 | 0 | 60'806 | 2'776 |
| Intervention arm | 142 | 7'083 | 17'781 | 1 | 180'640 | 2'834 |
| ***The Netherlands*** |  |  |  |  |  |  |
| Control arm | 210 | 4'788 | 6'688 | 3 | 64'430 | 2'888 |
| Intervention arm | 216 | 8'933 | 23'170 | 0 | 184'219 | 3'541 |

Note: Local costs expressed in Swiss Francs (CHF) using purchasing power parities.

Table 6S Costs of nursing homes per patient (CHF)

| **Costs of nursing homes (CHF)** | N | Mean | Std. Dev. | Min | Max | Median |
| --- | --- | --- | --- | --- | --- | --- |
|  |  |  |  |  |  |  |
| ***All countries*** |  |  |  |  |  |  |
| Control arm | 960 | 9'789 | 29'152 | 0 | 148'885 | 0 |
| Intervention arm | 900 | 8'699 | 27'089 | 0 | 145'115 | 0 |
| ***Switzerland*** |  |  |  |  |  |  |
| Control arm | 349 | 16'727 | 36'272 | 0 | 124'532 | 0 |
| Intervention arm | 432 | 12'689 | 31'543 | 0 | 138'669 | 0 |
| ***Ireland*** |  |  |  |  |  |  |
| Control arm | 202 | 9'691 | 30'408 | 0 | 136'720 | 0 |
| Intervention arm | 128 | 7'335 | 27'631 | 0 | 140'636 | 0 |
| ***Belgium*** |  |  |  |  |  |  |
| Control arm | 206 | 1'885 | 7'698 | 0 | 40'145 | 0 |
| Intervention arm | 137 | 2'290 | 9'023 | 0 | 41'236 | 0 |
| ***The Netherlands*** |  |  |  |  |  |  |
| Control arm | 203 | 5'981 | 25'024 | 0 | 148'885 | 0 |
| Intervention arm | 203 | 5'396 | 23'110 | 0 | 145'115 | 0 |

Note: Local costs expressed in Swiss Francs (CHF) using purchasing power parities.

Table 7S. Costs of nursing visits at home per patient (CHF)

| **Costs of nursing visits at home (CHF)** | N | Mean | Std. Dev. | Min | Max | Median |
| --- | --- | --- | --- | --- | --- | --- |
|  |  |  |  |  |  |  |
| ***All countries*** |  |  |  |  |  |  |
| Control arm | 986 | 5'951 | 21'227 | 0 | 263'668 | 0 |
| Intervention arm | 917 | 4'770 | 14'509 | 0 | 148'382 | 0 |
| ***Switzerland*** |  |  |  |  |  |  |
| Control arm | 361 | 6'294 | 21'785 | 0 | 176'717 | 0 |
| Intervention arm | 439 | 3'367 | 11'703 | 0 | 148'382 | 0 |
| ***Ireland*** |  |  |  |  |  |  |
| Control arm | 205 | 4'967 | 21'382 | 0 | 248'250 | 0 |
| Intervention arm | 131 | 3'060 | 8'093 | 0 | 52'810 | 0 |
| ***Belgium*** |  |  |  |  |  |  |
| Control arm | 214 | 3'088 | 19'171 | 0 | 263'668 | 0 |
| Intervention arm | 141 | 1'047 | 2'761 | 0 | 20'995 | 0 |
| ***The Netherlands*** |  |  |  |  |  |  |
| Control arm | 206 | 9'301 | 21'766 | 0 | 203'977 | 0 |
| Intervention arm | 206 | 11'396 | 23'270 | 0 | 147'765 | 0 |

Note: Local costs expressed in Swiss Francs (CHF) using purchasing power parities.

Table 8S. Opportunity costs of informal care per patient (CHF)

| **Opportunity costs of informal care (CHF)** | Obs | Mean | Std. Dev. | Min | Max | Median |
| --- | --- | --- | --- | --- | --- | --- |
|  |  |  |  |  |  |  |
| ***All countries*** |  |  |  |  |  |  |
| Control arm | 988 | 3'858 | 12'819 | 0 | 97'728 | 0 |
| Intervention arm | 917 | 2'390 | 9'967 | 0 | 97'728 | 0 |
| ***Switzerland*** |  |  |  |  |  |  |
| Control arm | 361 | 630 | 2'647 | 0 | 22'709 | 0 |
| Intervention arm | 439 | 285 | 1'169 | 0 | 11'155 | 0 |
| ***Ireland*** |  |  |  |  |  |  |
| Control arm | 206 | 14'553 | 24'066 | 0 | 97'728 | 0 |
| Intervention arm | 132 | 11'550 | 21'507 | 0 | 97'728 | 0 |
| ***Belgium*** |  |  |  |  |  |  |
| Control arm | 215 | 1'325 | 5'088 | 0 | 37'440 | 0 |
| Intervention arm | 141 | 547 | 1'441 | 0 | 10'742 | 0 |
| ***The Netherlands*** |  |  |  |  |  |  |
| Control arm | 206 | 1'464 | 5'185 | 0 | 34'310 | 0 |
| Intervention arm | 205 | 2'269 | 8'859 | 0 | 95'232 | 0 |

Note: Local costs expressed in Swiss Francs (CHF) using purchasing power parities.

Table 9S. Costs generated through time recording during medication reconciliation per patient (CHF)

| **Costs of time recording (mean)** | ***All*** | ***Switzerland*** | ***Ireland*** | ***Belgium*** | ***Netherlands*** |
| --- | --- | --- | --- | --- | --- |
| Control arm | 39 | 34 | 93 | 21 | 13 |
| Intervention arm | 103 | 87 | 147 | 15 | 99 |

Note: Local costs expressed in Swiss Francs (CHF) using purchasing power parities.

*5.2 heterogeneity test*

We performed between-country heterogeneity tests for qualitative and quantitative interaction on incremental QALYs and incremental costs. The qualitative interaction testing assessed whether the outcome of interest has different signs, while the quantitative interaction testing verified whether the treatment has equal signs but different magnitude (Cook JR, Drummond M, Glick H, Heyse JF. Assessing the appropriateness of combining economic data from multinational clinical trials. Stat Med. 2003;22(12):1955-76).

*Qualitative interaction test*

The null hypothesis was that the outcomes are all greater than 0 or all less than 0 for the 4 countries involved. We performed a likelihood ratio test according to the following formula:

Q = min (Q^-^,Q^+^) > c, where c is equal to 13.47 if the number of groups (countries) is 4 and:

$Q^{-}=\sum_{i=1}^{k} (D_{i}^{2}/S_{i}^{2})\forall D_{i}>0$ and $Q^{+}=\sum_{i=1}^{k} (D_{i}^{2}/S_{i}^{2})\forall D_{i}<0$

Where D is the difference of the outcomes between intervention and control patients, i is the country (from 1 to K) and S2 the variance of the difference in outcomes.

The value of Q for both outcomes (incremental QALYs and incremental costs) was equal to 0, meaning that the treatment effect was positive for some countries and negative for others. Thus, the test highlighted the presence of heterogeneity for both outcomes.

*Quantitative interaction test*

For the sake of completeness, we also performed quantitative interaction testing, by computing the following quantity:

$$H=\sum_{i-1}^{k} {(D_{i}-\overline{D)}}^{2}/S_{i}^{2}$$

Where $\overline{\boldsymbol{D}}\mathbf{=}\left[ \sum_{\boldsymbol{i}\mathbf{-1}}^{\boldsymbol{k}} \boldsymbol{D}_{\boldsymbol{i}}\mathbf{/}\boldsymbol{S}_{\boldsymbol{i}}^{\mathbf{2}} \right]\mathbf{/}\sum_{\boldsymbol{i}\mathbf{-1}}^{\boldsymbol{k}} \mathbf{1}\mathbf{/}\boldsymbol{S}_{\boldsymbol{i}}^{\mathbf{2}}$

The values of H were compared to the critical values of the X2 distribution with 3 (i.e.: 4 groups - 1) degrees of freedom which in our case was equal to 9.35.

We obtained H=5.6 for incremental QALYs and H=11.8 for incremental costs. In this case, the test rejected the null hypothesis of no difference in magnitude for incremental costs, but not for incremental QALYs.

*5.3 country-specific cost-effectiveness analyses*

Table 10S. Country specific cost-effectiveness analysis, costs expressed in CHF

|  | (1) | (2) | (3) | (4) |
| --- | --- | --- | --- | --- |
|  | Switzerland | Ireland | Belgium | Netherlands |
| **Effects on costs (CHF)** |  |  |  |  |
| Intervention Arm | -7'027* | -8'963 | -6'081 | 5'758 |
|  | [-13'130,-924] | [-20'373,2'445] | [-17'073,4'910] | [-5'273,16'789] |
| Age | 868*** | 616 | 790* | 1'348** |
|  | [358,1'378] | [-248,1'481] | [64,1'517] | [498,2'199] |
| Female | -4'868 | 1'991 | 3'198 | 5'276 |
|  | [-11'379,1'642] | [-8'214,12'197] | [-5'158,11'554] | [-4'560,15'113] |
| Utility 6 months before | 8'801 | -7'693 | -3'612 | -15'690 |
|  | [-18'496,36'099] | [-36'154,20'768] | [-24'192,16'966] | [-33'208,1'827] |
| Utility baseline | -21'009** | -22'473 | -7'956 | -24'159** |
|  | [-34'929,-7'090] | [-45'271,324] | [-26'265,10'351] | [-41'881,-6'438] |
| Number of drugs | 914** | 862 | 360 | 2'310*** |
|  | [226,1'603] | [-482,2'207] | [-1'039,1'761] | [1'115,3'505] |
| Number of comorbidities | 669** | 1'652* | 353 | -510 |
|  | [236,1'101] | [316,2'988] | [-769,1'476] | [-2'194,1'173] |
| Housebound | 8'388 | 5'605 | -144 | -1'339 |
|  | [-6'306,23'083] | [-8'029,19'241] | [-10'594,10'305] | [-15'453,12'775] |
| Smoker | 1'608 | 535 | 1'257 | -44 |
|  | [-9'382,12'598] | [-19'926,20'997] | [-18'259,20'775] | [-15'692,15'603] |
| High School | -804 | 2'558 | 7'626 | 5'556 |
|  | [-8'817,7'207] | [-9'902,15'020] | [-4'560,19'812] | [-4'963,16'076] |
| University | 3'246 | -6'967 | 9'208 | -4'525 |
|  | [-6'793,13'285] | [-21'521,7'585] | [-997,19'414] | [-19'141,10'091] |
| Living in nursing home | 52'550*** | 74'374*** | 31'888* | 47'957*** |
|  | [40'647,64'453] | [32'998,115'750] | [3'077,60'698] | [21'307,74'607] |
| Dementia | 263 | 12'100 | -6'629 | 35'857 |
|  | [-10'802,11'330] | [-9'880,34'081] | [-72'156,58'898] | [-32'201,103'915] |
| N. of hosp. 1 year before | 1'928* | 4'307* | 1'850 | 4'435** |
|  | [35,3'821] | [60,8'554] | [-1'939,5'639] | [1'441,7'429] |
| Medical ward | 4'184 | 29'638 | 8'355 | 5'829 |
|  | [-4'827,13'195] | [-13'973,73'250] | [-2'848,19'558] | [-4'030,15'690] |
| Observation time | 155*** | 116*** | 100*** | 202*** |
|  | [125,185] | [67,166] | [55,145] | [163,240] |
| Duration baseline hosp. | 926*** | 212 | 569** | 571** |
|  | [502,1'349] | [-91,515] | [137,1'002] | [199,943] |
| Constant | -100'425*** | -85'147 | -83'834** | -139'943*** |
|  | [-147'989,-52'861] | [-174'006,3'712] | [-145'515,-22'153] | [-219'992,-59'895] |
| **Effects on QALYs** |  |  |  |  |
| Intervention Arm | 0.006 | -0.006 | 0.023 | 0.074 |
|  | [-0.038,0.051] | [-0.072,0.059] | [-0.064,0.111] | [-0.002,0.151] |
| Age | -0.007*** | -0.006* | -0.003 | -0.008** |
|  | [-0.011,-0.003] | [-0.012,-0.001] | [-0.009,0.002] | [-0.013,-0.003] |
| Female | -0.015 | -0.045 | 0.012 | 0.026 |
|  | [-0.055,0.024] | [-0.105,0.014] | [-0.079,0.104] | [-0.043,0.096] |
| Utility 6 months before | 0.233** | 0.219* | 0.154 | 0.133* |
|  | [0.108,0.359] | [0.043,0.394] | [-0.090,0.398] | [0.022,0.243] |
| Utility baseline | 0.252*** | 0.362*** | 0.387** | 0.332*** |
|  | [0.164,0.340] | [0.230,0.494] | [0.130,0.644] | [0.219,0.444] |
| Number of drugs | -0.007** | -0.007 | -0.003 | -0.011* |
|  | [-0.012,-0.002] | [-0.016,0.001] | [-0.014,0.007] | [-0.020,-0.001] |
| Number of comorbidities | -0.002 | 0.002 | -0.007 | -0.004 |
|  | [-0.005,0.000] | [-0.006,0.012] | [-0.017,0.002] | [-0.016,0.008] |
| Housebound | -0.124* | -0.023 | -0.058 | -0.031 |
|  | [-0.219,-0.028] | [-0.106,0.060] | [-0.143,0.026] | [-0.150,0.086] |
| Smoker | -0.012 | 0.014 | 0.041 | 0.038 |
|  | [-0.081,0.056] | [-0.102,0.130] | [-0.070,0.154] | [-0.067,0.144] |
| High School | -0.013 | 0.000 | 0.019 | 0.007 |
|  | [-0.069,0.041] | [-0.081,0.083] | [-0.067,0.107] | [-0.066,0.082] |
| University | -0.052 | 0.023 | 0.051 | 0.116* |
|  | [-0.119,0.015] | [-0.063,0.111] | [-0.025,0.129] | [0.013,0.218] |
| Living in nursing home | 0.020 | -0.032 | 0.041 | -0.006 |
|  | [-0.060,0.101] | [-0.252,0.187] | [-0.171,0.254] | [-0.334,0.322] |
| Dementia | -0.004 | -0.016 | -0.032 | 0.027 |
|  | [-0.083,0.075] | [-0.143,0.110] | [-0.459,0.394] | [-0.664,0.720] |
| N. of hosp. 1 year before | -0.008 | -0.023 | -0.010 | -0.016 |
|  | [-0.021,0.004] | [-0.050,0.002] | [-0.045,0.024] | [-0.036,0.004] |
| Medical ward | -0.073* | -0.181 | -0.070 | -0.057 |
|  | [-0.138,-0.009] | [-0.452,0.088] | [-0.147,0.005] | [-0.123,0.009] |
| Duration baseline hosp. | -0.007*** | -0.003** | -0.000 | -0.004*** |
|  | [-0.011,-0.003] | [-0.004,-0.001] | [-0.003,0.002] | [-0.006,-0.001] |
| Constant | 1.201*** | 1.067*** | 0.701** | 1.176*** |
|  | [0.878,1.524] | [0.543,1.590] | [0.263,1.139] | [0.662,1.691] |
| Observations | 822 | 346 | 388 | 452 |

Note: GSEM models for Switzerland and Belgium, SUR models for Belgium and the Netherlands. 95% confidence intervals in brackets. * p<0.05, ** p<0.01, ***<0.001. Local costs are expressed in Swiss Francs (CHF) using purchasing power parities. We applied local costs converted to CHF through PPP to each country. German EQ-5D-5L valuation algorithm applied to Switzerland, UK EQ-5D-5L valuation algorithm applied to Ireland and Dutch EQ-5D-5L algorithm applied to Belgium and the Netherlands. KEY: hosp. = hospitalisations; N = number; QALY= quality adjusted life year.

*5.4 Subgroup cost-effectiveness analyses*

Table 11S. Subgroups by sex, costs expressed in CHF

|  | (1) | (2) |
| --- | --- | --- |
|  | Females | Males |
| **Effects on costs (CHF)** |  |  |
|  |  |  |
| Intervention Arm | -3'642 | -4'270 |
|  | [-9'983,2'699] | [-9'763,1'222] |
| Age | 1'053*** | 663** |
|  | [531,1'575] | [219,1'108] |
| Utility 6 months before | -2'750 | -5'556 |
|  | [-17'803,12'302] | [-22'680,11'568] |
| Utility baseline | -21'973** | -21'807** |
|  | [-34'834,-9'112] | [-34'069,-9'545] |
| Number of drugs | 1'243** | 984** |
|  | [438,2'048] | [331,1'636] |
| Number of comorbidities | 580 | 570* |
|  | [-23,1'183] | [76,1'064] |
| Housebound | -2'387 | 8'682* |
|  | [-11'640,6'865] | [39,17'324] |
| Smoker | -1'910 | 1'948 |
|  | [-15'139,11'318] | [-7'149,11'045] |
| High School | 4'129 | -1'645 |
|  | [-3'210,11'468] | [-8'876,5'585] |
| University | -860 | 494 |
|  | [-11'014,9'293] | [-7'378,8'367] |
| Living in nursing home | 42'872*** | 57'410*** |
|  | [30'074,55'669] | [42'012,72'808] |
| Dementia | 7'890 | -2'554 |
|  | [-6'930,22'710] | [-15'341,10'232] |
| N. of hosp. 1 year before | 3'945*** | 2'394** |
|  | [1'600,6'290] | [629,4'159] |
| Medical ward | 3'680 | 10'290** |
|  | [-5'739,13'100] | [3'030,17'549] |
| Observation time | 177*** | 135*** |
|  | [146,208] | [112,159] |
| Duration baseline hosp. | 531*** | 424*** |
|  | [264,797] | [175,673] |
| Ireland | 5'676 | -6'462 |
|  | [-4'132,15'484] | [-16'782,3'856] |
| Belgium | 302 | -13'949** |
|  | [-10'996,11'600] | [-23'390,-4'507] |
| Netherlands | 19'293*** | 1'854 |
|  | [9'131,29'455] | [-7'245,10'954] |
| Constant | -121'321*** | -65'205** |
|  | [-168'693,-73'949] | [-105'533,-24'878] |
| **Effects on QALYs** |  |  |
|  |  |  |
| Intervention Arm | 0.002 | 0.044* |
|  | [-0.040,0.045] | [0.006,0.083] |
| Age | -0.007*** | -0.005*** |
|  | [-0.010,-0.003] | [-0.008,-0.002] |
| Utility 6 months before | 0.168** | 0.204*** |
|  | [0.068,0.268] | [0.108,0.300] |
| Utility baseline | 0.347*** | 0.303*** |
|  | [0.249,0.446] | [0.244,0.362] |
| Number of drugs | -0.008** | -0.006** |
|  | [-0.013,-0.003] | [-0.011,-0.002] |
| Number of comorbidities | -0.002 | -0.003 |
|  | [-0.007,0.002] | [-0.006,0.000] |
| Housebound | -0.003 | -0.106*** |
|  | [-0.069,0.062] | [-0.163,-0.048] |
| Smoker | -0.012 | 0.033 |
|  | [-0.106,0.080] | [-0.024,0.092] |
| High School | 0.006 | 0.002 |
|  | [-0.042,0.055] | [-0.049,0.054] |
| University | 0.040 | 0.025 |
|  | [-0.027,0.108] | [-0.031,0.082] |
| Living in nursing home | 0.027 | -0.010 |
|  | [-0.070,0.125] | [-0.134,0.114] |
| Dementia | 0.040 | -0.029 |
|  | [-0.073,0.154] | [-0.115,0.054] |
| N. of hosp. 1 year before | -0.013 | -0.014* |
|  | [-0.028,0.001] | [-0.025,-0.002] |
| Medical ward | -0.068* | -0.067** |
|  | [-0.123,-0.013] | [-0.118,-0.017] |
| Duration baseline hosp. | -0.004*** | -0.003** |
|  | [-0.005,-0.002] | [-0.005,-0.001] |
| Ireland | -0.057 | 0.006 |
|  | [-0.130,0.014] | [-0.068,0.080] |
| Belgium | -0.050 | -0.024 |
|  | [-0.132,0.031] | [-0.084,0.036] |
| Netherlands | -0.036 | -0.054 |
|  | [-0.099,0.027] | [-0.115,0.006] |
| Constant | 1.096*** | 0.972*** |
|  | [0.773,1.418] | [0.720,1.224] |
| Observations | 898 | 1'110 |

Note: GSEM model for males, SUR model for females. 95% confidence intervals in brackets. * p<0.05, ** p<0.01, ***<0.001. Local costs expressed in Swiss Francs (CHF) using purchasing power parities. KEY: hosp. = hospitalisations; N = number; QALY= quality adjusted life year.

Table 12S. Subgroups analysis: community dwelling versus nursing homes. Costs expressed in CHF

|  | (1) | (2) |
| --- | --- | --- |
|  | Community dwelling | Nursing homes |
| **Effects on costs (CHF)** |  |  |
|  |  |  |
| Intervention Arm | -3'081 | -902 |
|  | [-7'344,1'182] | [-15'013,13'207] |
| Age | 849*** | -168 |
|  | [485,1'214] | [-1'246,909] |
| Female | 100 | -7'047 |
|  | [-4'286,4'487] | [-21'793,7'698] |
| Utility 6 months before | -5'799 | -5'287 |
|  | [-19'381,7'783] | [-30'229,19'655] |
| Utility baseline | -25'633*** | 8'976 |
|  | [-35'138,-16'127] | [-11'928,29'881] |
| Number of drugs | 1'084*** | 324 |
|  | [542,1'627] | [-1'119,1'767] |
| Number of comorbidities | 524* | 170 |
|  | [121,927] | [-957,1'298] |
| Housebound | 3'688 | -10'921 |
|  | [-2'965,10'343] | [-29'078,7'236] |
| Smoker | -319 | 13'963 |
|  | [-8'257,7'618] | [-9'398,37'325] |
| High School | 1'133 | -2'406 |
|  | [-4'255,6'521] | [-19'028,14'216] |
| University | 1'640 | 14'986 |
|  | [-4'575,7'857] | [-7'772,37'744] |
| Dementia | 5'531 | -16'560 |
|  | [-5'014,16'077] | [-35'129,2'007] |
| N. of hosp. 1 year before | 2'861*** | 1'589 |
|  | [1'313,4'408] | [-2'792,5'970] |
| Medical ward | 9'268** | -10'178 |
|  | [3'383,15'153] | [-30'688,10'332] |
| Observation time | 135*** | 357*** |
|  | [115,155] | [284,431] |
| Duration baseline hosp. | 444*** | 1'195* |
|  | [262,626] | [26,2'364] |
| Ireland | -654 | 33'398* |
|  | [-8'218,6'910] | [427,66'370] |
| Belgium | -9'099* | -46'814*** |
|  | [-16'678,-1'519] | [-74'327,-19'302] |
| Netherlands | 8'209* | 18'151 |
|  | [1'024,15'393] | [-7'171,43'473] |
| Constant | -82'826*** | 3'691 |
|  | [-115'980,-49'671] | [-106'875,114'259] |
| **Effects on QALYs** |  |  |
|  |  |  |
| Intervention Arm | 0.023 | 0.068 |
|  | [-0.001,0.048] | [-0.051,0.189] |
| Age | -0.007*** | -0.004 |
|  | [-0.009,-0.004] | [-0.013,0.003] |
| Female | -0.009 | 0.068 |
|  | [-0.036,0.018] | [-0.048,0.186] |
| Utility 6 months before | 0.199*** | 0.172 |
|  | [0.140,0.258] | [-0.032,0.376] |
| Utility baseline | 0.300*** | 0.329*** |
|  | [0.256,0.344] | [0.149,0.508] |
| Number of drugs | -0.007*** | -0.004 |
|  | [-0.011,-0.004] | [-0.017,0.007] |
| Number of comorbidities | -0.002* | 0.003 |
|  | [-0.005,-0.000] | [-0.005,0.012] |
| Housebound | -0.063** | -0.103 |
|  | [-0.102,-0.025] | [-0.255,0.049] |
| Smoker | 0.017 | 0.046 |
|  | [-0.028,0.064] | [-0.139,0.231] |
| High School | 0.012 | -0.098 |
|  | [-0.020,0.044] | [-0.225,0.028] |
| University | 0.017 | -0.013 |
|  | [-0.020,0.055] | [-0.194,0.168] |
| Dementia | 0.012 | -0.052 |
|  | [-0.052,0.076] | [-0.204,0.098] |
| N. of hosp. 1 year before | -0.019*** | 0.021 |
|  | [-0.028,-0.009] | [-0.019,0.061] |
| Medical ward | -0.077*** | 0.029 |
|  | [-0.113,-0.041] | [-0.147,0.206] |
| Duration baseline hosp. | -0.003*** | -0.003 |
|  | [-0.005,-0.002] | [-0.014,0.006] |
| Ireland | -0.026 | -0.052 |
|  | [-0.068,0.015] | [-0.309,0.205] |
| Belgium | -0.036 | 0.025 |
|  | [-0.081,0.008] | [-0.191,0.241] |
| Netherlands | -0.056** | -0.099 |
|  | [-0.098,-0.013] | [-0.311,0.113] |
| Constant | 1.109*** | 0.664 |
|  | [0.903,1.315] | [-0.190,1.518] |
| Observations | 1'766 | 99 |

Note: GSEM model for community dwelling, SUR model for nursing homes. 95% confidence intervals in brackets. * p<0.05, ** p<0.01, ***<0.001. Local costs expressed in Swiss Francs (CHF) using purchasing power parities. KEY: hosp. = hospitalisations; N = number; QALY= quality adjusted life year.

Table 13S. Subgroups analysis: medical versus surgical ward. Costs expressed in CHF

|  | (1) | (2) |
| --- | --- | --- |
|  | Medical ward | Surgical ward |
| **Effects on costs (CHF)** |  |  |
|  |  |  |
| Intervention Arm | -4'615 | -1'046 |
|  | [-9'719,488] | [-10'463,8'370] |
| Age | 865*** | 888* |
|  | [471,1'259] | [100,1'676] |
| Female | -2'223 | 7'016 |
|  | [-7'036,2'589] | [-2'178,16'212] |
| Utility 6 months before | -5'879 | -261 |
|  | [-23'713,11'955] | [-17'154,16'632] |
| Utility baseline | -21'326** | -24'479*** |
|  | [-35'162,-7'489] | [-37'795,-11'163] |
| Number of drugs | 1'053*** | 1'233* |
|  | [477,1'629] | [21,2'446] |
| Number of comorbidities | 579** | 794 |
|  | [166,992] | [-141,1'730] |
| Housebound | 5'364 | -3'301 |
|  | [-1'681,12'410] | [-18'149,11'547] |
| Smoker | -1'770 | 11'483 |
|  | [-10'729,7'188] | [-3'457,26'424] |
| High School | 2'768 | -5'587 |
|  | [-2'839,8'376] | [-17'543,6'369] |
| University | 1'405 | -1'946 |
|  | [-5'349,8'159] | [-15'017,11'124] |
| Living in Nursing Home | 46'489*** | 62'069*** |
|  | [36'006,56'972] | [38'891,85'248] |
| Dementia | 4'069 | -3'407 |
|  | [-6'107,14'245] | [-40'564,33'750] |
| N. of hosp. 1 year before | 3'173*** | 2'785 |
|  | [1'575,4'770] | [-255,5'825] |
| Observation time | 174*** | 153*** |
|  | [149,199] | [109,197] |
| Duration baseline hosp. | 482*** | 643** |
|  | [280,683] | [231,1'055] |
| Ireland | 407 | -25'928 |
|  | [-6'487,7'302] | [-62'919,11'062] |
| Belgium | -6'699 | -13'301 |
|  | [-14'857,1'457] | [-26'960,357] |
| Netherlands | 12'031** | 4'680 |
|  | [4'489,19'573] | [-7'533,16'894] |
| Constant | -90'823*** | -97'973** |
|  | [-125'975,-55'671] | [-166'681,-29'265] |
| **Effects on QALYs** |  |  |
|  |  |  |
| Intervention Arm | 0.019 | 0.043 |
|  | [-0.010,0.049] | [-0.020,0.108] |
| Age | -0.006*** | -0.004 |
|  | [-0.008,-0.004] | [-0.010,0.001] |
| Female | -0.001 | -0.003 |
|  | [-0.036,0.032] | [-0.071,0.064] |
| Utility 6 months before | 0.189*** | 0.162 |
|  | [0.114,0.265] | [-0.015,0.340] |
| Utility baseline | 0.347*** | 0.234*** |
|  | [0.284,0.410] | [0.157,0.310] |
| Number of drugs | -0.006** | -0.013** |
|  | [-0.009,-0.002] | [-0.022,-0.005] |
| Number of comorbidities | -0.002 | -0.005 |
|  | [-0.005,0.000] | [-0.012,0.000] |
| Housebound | -0.061** | -0.029 |
|  | [-0.108,-0.015] | [-0.135,0.075] |
| Smoker | 0.017 | 0.015 |
|  | [-0.036,0.072] | [-0.085,0.116] |
| High School | -0.000 | 0.011 |
|  | [-0.043,0.043] | [-0.064,0.086] |
| University | 0.027 | 0.012 |
|  | [-0.020,0.074] | [-0.081,0.107] |
| Living in Nursing Home | 0.038 | -0.096 |
|  | [-0.043,0.120] | [-0.339,0.146] |
| Dementia | -0.007 | 0.023 |
|  | [-0.088,0.073] | [-0.206,0.253] |
| N. of hosp. 1 year before | -0.014** | -0.008 |
|  | [-0.024,-0.004] | [-0.029,0.011] |
| Duration baseline hosp. | -0.003*** | -0.003** |
|  | [-0.005,-0.002] | [-0.006,-0.001] |
| Ireland | -0.025 | 0.055 |
|  | [-0.085,0.035] | [-0.183,0.293] |
| Belgium | -0.028 | -0.089 |
|  | [-0.084,0.028] | [-0.183,0.004] |
| Netherlands | -0.031 | -0.108** |
|  | [-0.078,0.015] | [-0.181,-0.035] |
| Constant | 0.932*** | 1.083*** |
|  | [0.707,1.158] | [0.665,1.500] |
| Observations | 1'589 | 419 |

Note: SUR models. 95% confidence intervals in brackets. * p<0.05, ** p<0.01, ***<0.001. Local costs expressed in Swiss Francs (CHF) using purchasing power parities. KEY: hosp. = hospitalisations; N = number; QALY= quality adjusted life year.

Table 14S. Subgroups analysis by age groups. Costs expressed in CHF

|  | (1) | (2) | (3) |
| --- | --- | --- | --- |
|  | Age 70-79 | Age 80-89 | Age 90 or more |
| **Effects on costs (CHF)** |  |  |  |
|  |  |  |  |
| Intervention Arm | -2'547 | -5'293 | 984 |
|  | [-7'740,2'646] | [-12'497,1'909] | [-13'668,15'637] |
| Age | 233 | 1'143 | -207 |
|  | [-696,1'163] | [-214,2'501] | [-3'266,2'852] |
| Female | -1'406 | 1'305 | -6'913 |
|  | [-6'736,3'924] | [-5'786,8'396] | [-21'648,7'821] |
| Utility 6 months before | -10'780 | 5'160 | 2'423 |
|  | [-27'047,5'485] | [-11'753,22'074] | [-24'117,28'964] |
| Utility baseline | -15'563** | -28'375*** | -26'048* |
|  | [-26'494,-4'633] | [-42'554,-14'196] | [-48'361,-3'735] |
| Number of drugs | 1'740*** | 483 | -1'236 |
|  | [1'093,2'386] | [-403,1'370] | [-3'016,544] |
| Number of comorbidities | 655* | 371 | 734 |
|  | [151,1'159] | [-287,1'030] | [-409,1'878] |
| Housebound | -4'878 | 11'265* | -528 |
|  | [-14'437,4'680] | [572,21'958] | [-18'730,17'674] |
| Smoker | 3'470 | -2'118 | -61'285* |
|  | [-4'976,11'917] | [-18'964,14'727] | [-115'476,-7'094] |
| High School | 3'700 | -2'209 | -2'953 |
|  | [-2'920,10'320] | [-11'230,6'812] | [-18'130,12'224] |
| University | 3'558 | -1'615 | 3'466 |
|  | [-3'994,11'110] | [-11'931,8'699] | [-16'599,23'531] |
| Living in Nursing Home | 64'071*** | 45'327*** | 41'351*** |
|  | [44'858,83'284] | [29'657,60'997] | [21'873,60'829] |
| Dementia | 7'340 | 3'435 | 3'962 |
|  | [-7'666,22'347] | [-11'290,18'162] | [-20'638,28'562] |
| N. of hosp. 1 year before | 3'254*** | 1'975 | -240 |
|  | [1'533,4'975] | [-929,4'880] | [-4'351,3'870] |
| Medical ward | 4'931 | 11'800* | 4'290 |
|  | [-1'900,11'763] | [1'642,21'958] | [-19'203,27'784] |
| Observation time | 114*** | 159*** | 197*** |
|  | [89,139] | [129,188] | [149,245] |
| Duration baseline hosp. | 294* | 677*** | 410 |
|  | [52,535] | [374,980] | [-159,980] |
| Ireland | 5'920 | -8'848 | -8'355 |
|  | [-3'461,15'302] | [-20'060,2'364] | [-31'540,14'828] |
| Belgium | -4'771 | -12'390 | -26'279* |
|  | [-13'737,4'194] | [-25'222,440] | [-50'936,-1'621] |
| Netherlands | 8'649* | 9'138 | 13'342 |
|  | [454,16'845] | [-2'431,20'708] | [-8'905,35'590] |
| Constant | -40'942 | -110'989 | 25'575 |
|  | [-113'327,31'441] | [-228'178,6'200] | [-252'154,303'304] |
| **Effects on QALYs** |  |  |  |
|  |  |  |  |
| Intervention Arm | -0.000 | 0.063** | 0.047 |
|  | [-0.041,0.039] | [0.020,0.105] | [-0.078,0.173] |
| Age | -0.000 | -0.002 | -0.006 |
|  | [-0.007,0.006] | [-0.010,0.005] | [-0.032,0.019] |
| Female | 0.004 | -0.013 | 0.004 |
|  | [-0.035,0.043] | [-0.067,0.040] | [-0.130,0.139] |
| Utility 6 months before | 0.214*** | 0.174** | 0.134 |
|  | [0.127,0.301] | [0.069,0.279] | [-0.144,0.412] |
| Utility baseline | 0.276*** | 0.345*** | 0.351** |
|  | [0.182,0.369] | [0.252,0.439] | [0.144,0.559] |
| Number of drugs | -0.003 | -0.013*** | -0.007 |
|  | [-0.007,0.001] | [-0.019,-0.008] | [-0.025,0.009] |
| Number of comorbidities | -0.003* | -0.003 | 0.001 |
|  | [-0.007,-0.000] | [-0.007,0.000] | [-0.008,0.011] |
| Housebound | -0.046 | -0.061 | -0.066 |
|  | [-0.117,0.024] | [-0.128,0.006] | [-0.242,0.109] |
| Smoker | 0.012 | 0.013 | 0.222 |
|  | [-0.047,0.073] | [-0.081,0.109] | [-0.265,0.708] |
| High School | 0.004 | 0.002 | 0.059 |
|  | [-0.046,0.054] | [-0.053,0.057] | [-0.070,0.190] |
| University | 0.026 | 0.028 | 0.079 |
|  | [-0.032,0.085] | [-0.031,0.088] | [-0.116,0.276] |
| Living in Nursing Home | 0.016 | -0.043 | 0.180 |
|  | [-0.122,0.156] | [-0.152,0.065] | [-0.030,0.390] |
| Dementia | 0.005 | 0.014 | -0.072 |
|  | [-0.114,0.124] | [-0.090,0.118] | [-0.309,0.164] |
| N. of hosp. 1 year before | -0.016** | -0.013 | 0.003 |
|  | [-0.029,-0.004] | [-0.028,0.002] | [-0.037,0.044] |
| Medical ward | -0.056* | -0.111** | 0.084 |
|  | [-0.103,-0.008] | [-0.182,-0.040] | [-0.122,0.291] |
| Duration baseline hosp. | -0.004*** | -0.003*** | -0.001 |
|  | [-0.006,-0.002] | [-0.005,-0.001] | [-0.006,0.003] |
| Ireland | -0.023 | -0.060 | 0.116 |
|  | [-0.088,0.040] | [-0.153,0.031] | [-0.098,0.331] |
| Belgium | -0.031 | -0.065 | 0.069 |
|  | [-0.088,0.026] | [-0.148,0.016] | [-0.177,0.315] |
| Netherlands | -0.025 | -0.107* | 0.099 |
|  | [-0.078,0.027] | [-0.192,-0.022] | [-0.137,0.337] |
| Constant | 0.583* | 0.822* | 0.692 |
|  | [0.081,1.085] | [0.144,1.499] | [-1.686,3.070] |
| Observations | 1078 | 782 | 148 |

Note: GSEM models. 95% confidence intervals in brackets. * p<0.05, ** p<0.01, ***<0.001. Local costs expressed in Swiss Francs (CHF) using purchasing power parities.

Table 15S. Subgroups analysis by number of drugs taken. Costs expressed in CHF

|  | (1) | (2) |
| --- | --- | --- |
|  | N. of drugs: 5 to 9 | N. of drugs: 10 or more |
| **Effects on costs (CHF)** |  |  |
|  |  |  |
| Intervention Arm | -3'884 | -3'177 |
|  | [-10'667,2'899] | [-8'548,2'192] |
| Age | 1'256*** | 565* |
|  | [709,1'803] | [130,1'000] |
| Female | 2'538 | -2'086 |
|  | [-4'125,9'202] | [-7'282,3'109] |
| Utility 6 months before | -5'583 | -3'913 |
|  | [-22'291,11'125] | [-20'033,12'206] |
| Utility baseline | -23'405*** | -21'940** |
|  | [-35'158,-11'652] | [-34'843,-9'038] |
| Number of drugs | 2'234 | 1'313*** |
|  | [-143,4'612] | [593,2'033] |
| Number of comorbidities | 606 | 630** |
|  | [-134,1'346] | [192,1'067] |
| Housebound | 5'214 | 698 |
|  | [-4'958,15'386] | [-7'438,8'835] |
| Smoker | 1'169 | 2'765 |
|  | [-11'069,13'407] | [-7'396,12'927] |
| High School | 3'124 | -225 |
|  | [-5'444,11'692] | [-6'486,6'035] |
| University | 6'074 | -4'234 |
|  | [-3'331,15'480] | [-12'170,3'701] |
| Living in Nursing Home | 42'601** | 50'943*** |
|  | [17'007,68'195] | [40'191,61'695] |
| Dementia | 10'588 | -424 |
|  | [-6'741,27'919] | [-12'583,11'734] |
| N. of hosp. 1 year before | 1'893 | 3'266*** |
|  | [-799,4'586] | [1'623,4'908] |
| Medical ward | 7'019 | 7'412 |
|  | [-1'270,15'308] | [-275,15'101] |
| Observation time | 107*** | 195*** |
|  | [78,136] | [168,222] |
| Duration baseline hosp. | 596*** | 491*** |
|  | [295,897] | [272,710] |
| Ireland | -2'677 | 1'862 |
|  | [-14'431,9'076] | [-6'353,10'078] |
| Belgium | -7'129 | -9'339 |
|  | [-18'353,4'095] | [-18'807,128] |
| Netherlands | 1'537 | 15'479*** |
|  | [-9'529,12'605] | [7'536,23'423] |
| Constant | -116'786*** | -85'687*** |
|  | [-169'252,-64'320] | [-125'572,-45'802] |
| **Effects on QALYs** |  |  |
|  |  |  |
| Intervention Arm | 0.008 | 0.037* |
|  | [-0.035,0.052] | [0.002,0.073] |
| Age | -0.004* | -0.007*** |
|  | [-0.007,-0.000] | [-0.010,-0.004] |
| Female | 0.003 | -0.001 |
|  | [-0.047,0.054] | [-0.040,0.036] |
| Utility 6 months before | 0.215*** | 0.178*** |
|  | [0.124,0.305] | [0.096,0.258] |
| Utility baseline | 0.316*** | 0.320*** |
|  | [0.239,0.393] | [0.258,0.382] |
| Number of drugs | -0.010 | -0.007* |
|  | [-0.028,0.007] | [-0.013,-0.001] |
| Number of comorbidities | -0.001 | -0.003* |
|  | [-0.006,0.003] | [-0.006,-0.000] |
| Housebound | -0.093** | -0.029 |
|  | [-0.161,-0.025] | [-0.087,0.029] |
| Smoker | 0.054 | -0.012 |
|  | [-0.017,0.127] | [-0.077,0.052] |
| High School | 0.005 | -0.000 |
|  | [-0.048,0.059] | [-0.046,0.045] |
| University | 0.015 | 0.039 |
|  | [-0.044,0.076] | [-0.020,0.098] |
| Living in Nursing Home | 0.027 | 0.008 |
|  | [-0.207,0.262] | [-0.064,0.081] |
| Dementia | -0.005 | -0.001 |
|  | [-0.140,0.130] | [-0.081,0.078] |
| N. of hosp. 1 year before | -0.033*** | -0.007 |
|  | [-0.049,-0.017] | [-0.018,0.003] |
| Medical ward | -0.078** | -0.053* |
|  | [-0.129,-0.027] | [-0.106,-0.001] |
| Duration baseline hosp. | -0.003** | -0.003*** |
|  | [-0.006,-0.000] | [-0.005,-0.002] |
| Ireland | -0.006 | -0.042 |
|  | [-0.079,0.067] | [-0.108,0.022] |
| Belgium | -0.012 | -0.051 |
|  | [-0.076,0.052] | [-0.120,0.016] |
| Netherlands | -0.013 | -0.079** |
|  | [-0.104,0.078] | [-0.135,-0.024] |
| Constant | 0.859*** | 1.153*** |
|  | [0.559,1.160] | [0.903,1.403] |
| Observations | 807 | 1'201 |

Note: GSEM model for 5-9 drugs category, SUR model for 10 or more drugs. 95% confidence intervals in brackets. * p<0.05, ** p<0.01, ***<0.001. Local costs expressed in Swiss Francs (CHF) using purchasing power parities. KEY: hosp. = hospitalisations; N = number; QALY= quality adjusted life year.

Table 16S. Subgroups analysis by number of comorbidities. Costs expressed in CHF

|  | (1) | (2) |
| --- | --- | --- |
|  | N. comorbidities: 3-6 | N. comorbidities: 7 or more |
| **Effects on costs (CHF)** |  |  |
|  |  |  |
| Intervention Arm | 4'388 | -5'964* |
|  | [-6'349,15'127] | [-10'811,-1'117] |
| Age | 1'034* | 788*** |
|  | [184,1'884] | [391,1'186] |
| Female | 1'336 | -684 |
|  | [-8'731,11'404] | [-5'524,4'156] |
| Utility 6 months before | 709 | -3'173 |
|  | [-24'026,25'444] | [-18'155,11'808] |
| Utility baseline | -12'358 | -20'938*** |
|  | [-29'371,4'654] | [-31'317,-10'559] |
| Number of drugs | 1'224 | 1'135*** |
|  | [-253,2'702] | [573,1'698] |
| Number of comorbidities | -997 | 651** |
|  | [-6'566,4'572] | [211,1'091] |
| Housebound | 18'738* | 957 |
|  | [2'419,35'057] | [-6'696,8'612] |
| Smoker | 6'009 | 2'430 |
|  | [-9'767,21'786] | [-6'579,11'441] |
| High School | -5 | -236 |
|  | [-11'021,11'009] | [-6'247,5'773] |
| University | 1'440 | -205 |
|  | [-12'594,15'476] | [-7'313,6'902] |
| Living in Nursing Home | 58'136 | 49'775*** |
|  | [-11'762,128'035] | [38'872,60'677] |
| Dementia | 33'777* | -114 |
|  | [2'468,65'085] | [-10'782,10'552] |
| N. of hosp. 1 year before | 5'695* | 2'956*** |
|  | [832,10'557] | [1'341,4'571] |
| Medical ward | 20'413*** | 5'408 |
|  | [8'510,32'316] | [-1'626,12'443] |
| Observation time | 144*** | 178*** |
|  | [100,188] | [150,206] |
| Duration baseline hosp. | 422* | 606*** |
|  | [88,755] | [366,847] |
| Ireland | -214 | -607 |
|  | [-19'502,19'073] | [-8'109,6'893] |
| Belgium | 1'889 | -6'173 |
|  | [-19'278,23'058] | [-14'280,1'933] |
| Netherlands | 21'066* | 10'906** |
|  | [2'742,39'390] | [2'877,18'936] |
| Constant | -129'271** | -94'751*** |
|  | [-210'954,-47'587] | [-130'724,-58'777] |
| **Effects on QALYs** |  |  |
|  |  |  |
| Intervention Arm | -0.029 | 0.033* |
|  | [-0.105,0.046] | [0.004,0.063] |
| Age | -0.009** | -0.006*** |
|  | [-0.016,-0.003] | [-0.009,-0.003] |
| Female | -0.059 | 0.010 |
|  | [-0.187,0.067] | [-0.022,0.042] |
| Utility 6 months before | 0.062 | 0.209*** |
|  | [-0.129,0.254] | [0.128,0.289] |
| Utility baseline | 0.290*** | 0.326*** |
|  | [0.164,0.415] | [0.268,0.383] |
| Number of drugs | -0.007 | -0.006** |
|  | [-0.019,0.004] | [-0.010,-0.002] |
| Number of comorbidities | 0.015 | -0.003* |
|  | [-0.023,0.053] | [-0.006,-0.000] |
| Housebound | -0.060 | -0.051* |
|  | [-0.221,0.100] | [-0.095,-0.007] |
| Smoker | -0.019 | 0.009 |
|  | [-0.149,0.109] | [-0.048,0.066] |
| High School | 0.005 | 0.003 |
|  | [-0.075,0.085] | [-0.035,0.042] |
| University | 0.043 | 0.021 |
|  | [-0.064,0.151] | [-0.024,0.066] |
| Living in Nursing Home | 0.171 | 0.009 |
|  | [-0.221,0.562] | [-0.076,0.094] |
| Dementia | -0.046 | 0.005 |
|  | [-0.279,0.186] | [-0.073,0.084] |
| N. of hosp. 1 year before | -0.030 | -0.012* |
|  | [-0.076,0.016] | [-0.022,-0.002] |
| Medical ward | -0.041 | -0.062** |
|  | [-0.147,0.063] | [-0.109,-0.016] |
| Duration baseline hosp. | -0.003** | -0.003*** |
|  | [-0.006,-0.001] | [-0.005,-0.002] |
| Ireland | -0.034 | -0.020 |
|  | [-0.194,0.126] | [-0.073,0.032] |
| Belgium | 0.012 | -0.039 |
|  | [-0.140,0.164] | [-0.088,0.009] |
| Netherlands | -0.009 | -0.072** |
|  | [-0.139,0.121] | [-0.120,-0.024] |
| Constant | 1.348*** | 0.989*** |
|  | [0.713,1.983] | [0.735,1.242] |
| Observations | 310 | 1'513 |

Note: GSEM model for 3-6 comorbidities category, SUR model for 7 or more drugs. 95% confidence intervals in brackets. * p<0.05, ** p<0.01, ***<0.001. Local costs expressed in Swiss Francs (CHF) using purchasing power parities. KEY: hosp. = hospitalisations; N = number; QALY= quality adjusted life year.

*5.5 robustness checks*

Table 17S. Non-adjusted differences in mean costs and QALYs. Costs expressed in CHF

| Costs | -1'486 |
| --- | --- |
|  | [-6'153,3'180] |
| QALYs | 0.026 |
|  | [-0.005,0.057] |
| Observations | 2008 |

Note: Local costs expressed in Swiss Francs (CHF) using purchasing power parities.

Table 18S. GSEM analysis with gamma distributed errors of costs with a log link function. Costs expressed in CHF

| Costs | -5'154 |
| --- | --- |
|  | [-10'505 196] |
| QALYs | 0.025 |
|  | [-0.001,0.052] |
| Observations | 2008 |

Note: Local costs expressed in Swiss Francs (CHF) using purchasing power parities. Results represent the marginal effects.

Table 19S. Results from different regression models. Costs expressed in CHF

|  | (1) | (2) | (3) |
| --- | --- | --- | --- |
|  | SUR | Mixed | SUR, only observed |
| **Effects on costs (CHF)** |  |  |  |
|  |  |  |  |
| Intervention Arm | -3'822 | -3'670 | -4'022 |
|  | [-7'970,326] | [-7'662,321] | [-8'404,360] |
| Age | 864*** | 799*** | 754*** |
|  | [515,1'214] | [491,1'107] | [383,1'126] |
| Female | -141 | -64 | -2'276 |
|  | [-4'250,3'968] | [-4'502,4'374] | [-6'777,2'225] |
| Utility 6 months before | -4'496 | -3'977 | -1'682 |
|  | [-18'248,9'255] | [-18'007,10'053] | [-12'469,9'105] |
| Utility baseline | -22'049*** | -20'950*** | -27'633*** |
|  | [-32'355,-11'743] | [-31'095,-10'805] | [-36'061,-19'205] |
| Number of drugs | 1'087*** | 1'073*** | 1'098*** |
|  | [579,1'595] | [447,1'698] | [548,1'649] |
| Number of comorbidities | 607** | 591** | 552** |
|  | [230,985] | [223,958] | [140,965] |
| Housebound | 3'721 | 3'213 | 5'873 |
|  | [-2'925,10'369] | [-3'548,9'975] | [-1'011,12'758] |
| Smoker | 1'228 | 1'100 | -2'603 |
|  | [-6'491,8'947] | [-7'676,9'878] | [-10'691,5'484] |
| High School | 1'313 | 1'437 | -2'306 |
|  | [-3'745,6'372] | [-3'933,6'807] | [-7'772,3'159] |
| University | 1'290 | 1'468 | 709 |
|  | [-4'678,7'258] | [-4'562,7'499] | [-5'781,7'201] |
| Living in Nursing Home | 48'604*** | 49'571*** | 53'605*** |
|  | [39'109,58'099] | [36'424,62'717] | [42'082,65'128] |
| Dementia | 3'279 | 3'460 | -5'508 |
|  | [-6'436,12'995] | [-8'353,15'275] | [-17'148,6'130] |
| N. of hosp. 1 year before | 3'050*** | 2'867*** | 3'238*** |
|  | [1'615,4'485] | [1'417,4'316] | [1'659,4'817] |
| Medical ward | 8'275** | 7'760** | 8'311** |
|  | [2'894,13'656] | [2'444,13'077] | [2'463,14'159] |
| Observation time | 172*** | 141*** | 191*** |
|  | [149,195] | [121,161] | [171,212] |
| Duration baseline hosp. | 530*** | 488*** | 606*** |
|  | [351,708] | [285,692] | [375,836] |
| Ireland | -740 | -188 | -4'070 |
|  | [-7'264,5'783] | [-7'088,6'711] | [-11'054,2'913] |
| Belgium | -7'232* | -7'726* | -11'882** |
|  | [-14'200,-264] | [-15'225,-226] | [-19'130,-4'635] |
| Netherlands | 10'378** | 9'372** | 5'279 |
|  | [4'174,16'583] | [2'440,16'304] | [-1'682,12'241] |
| Constant | -100'322*** | -85'230*** | -93'668*** |
|  | [-131'169,-69'475] | [-117'571,-52'888] | [-128'204,-59'132] |
| **Effects on QALYs** |  |  |  |
|  |  |  |  |
| Intervention Arm | 0.025 | 0.024 | 0.017 |
|  | [-0.001,0.052] | [-0.002,0.052] | [-0.007,0.043] |
| Age | -0.006*** | -0.006*** | -0.007*** |
|  | [-0.008,-0.004] | [-0.008,-0.004] | [-0.009,-0.005] |
| Female | -0.000 | -0.000 | -0.004 |
|  | [-0.035,0.035] | [-0.037,0.036] | [-0.031,0.021] |
| Utility 6 months before | 0.191*** | 0.192*** | 0.181*** |
|  | [0.131,0.251] | [0.132,0.251] | [0.118,0.244] |
| Utility baseline | 0.316*** | 0.316*** | 0.309*** |
|  | [0.264,0.367] | [0.257,0.375] | [0.260,0.358] |
| Number of drugs | -0.007*** | -0.007*** | -0.007*** |
|  | [-0.010,-0.004] | [-0.010,-0.004] | [-0.010,-0.004] |
| Number of comorbidities | -0.003* | -0.003* | -0.001 |
|  | [-0.005,-0.000] | [-0.005,-0.000] | [-0.004,0.000] |
| Housebound | -0.056* | -0.056* | -0.080*** |
|  | [-0.099,-0.012] | [-0.098,-0.013] | [-0.120,-0.039] |
| Smoker | 0.015 | 0.016 | 0.036 |
|  | [-0.033,0.064] | [-0.034,0.066] | [-0.011,0.083] |
| High School | 0.003 | 0.004 | 0.003 |
|  | [-0.033,0.039] | [-0.032,0.041] | [-0.028,0.035] |
| University | 0.027 | 0.027 | -0.003 |
|  | [-0.015,0.069] | [-0.015,0.071] | [-0.041,0.034] |
| Living in Nursing Home | 0.013 | 0.013 | -0.000 |
|  | [-0.067,0.095] | [-0.067,0.094] | [-0.068,0.066] |
| Dementia | -0.006 | -0.006 | -0.008 |
|  | [-0.082,0.069] | [-0.078,0.065] | [-0.076,0.059] |
| N. of hosp. 1 year before | -0.014** | -0.013** | -0.021*** |
|  | [-0.023,-0.004] | [-0.022,-0.004] | [-0.030,-0.012] |
| Medical ward | -0.063*** | -0.064** | -0.099*** |
|  | [-0.099,-0.026] | [-0.103,-0.026] | [-0.133,-0.064] |
| Duration baseline hosp. | -0.003*** | -0.003*** | -0.005*** |
|  | [-0.005,-0.002] | [-0.005,-0.002] | [-0.006,-0.003] |
| Ireland | -0.030 | -0.029 | -0.010 |
|  | [-0.083,0.023] | [-0.084,0.026] | [-0.051,0.030] |
| Belgium | -0.036 | -0.036 | -0.024 |
|  | [-0.084,0.010] | [-0.079,0.007] | [-0.067,0.017] |
| Netherlands | -0.052* | -0.051* | -0.062** |
|  | [-0.092,-0.011] | [-0.101,-0.001] | [-0.103,-0.021] |
| Constant | 1.035*** | 1.035*** | 1.189*** |
|  | [0.841,1.230] | [0.834,1.236] | [0.997,1.380] |
| Observations | 2'008 | 2'008 | 1'449 |

Note: SUR model in column 1, linear mixed model (not simultaneous) in column 2, SUR model only on observed data in column 3. 95% confidence intervals in brackets. * p<0.05, ** p<0.01, ***<0.001. Local costs expressed in Swiss Francs (CHF) using purchasing power parities.

*5.6 Deterministic sensitivity analysis*

Table 20S. Results of the deterministic sensitivity analysis. Costs expressed in CHF

|  | Incremental cost with -30% of the specific cost | Incremental cost with +30% of the specific cost |
| --- | --- | --- |
|  |  |  |
| **Baseline** | -3’588 | -3’588 |
|  | [-7’716 540] | [-7’716 540] |
| Hospitalization costs | -3’209 | -3’966 |
|  | [-7011 592] | [ -8’488 556] |
| Rehabilitation costs | -3’659 | -3’523 |
|  | [-7’566 248] | [-7’915 868] |
| Medical visit costs | -3’617 | -3’558 |
|  | [-7’740 505] | [-7’692 574] |
| Nursing home’ costs | -3’027 | -4’155 |
|  | [-6’956 902] | [-8’555 244] |
| Nursing at home costs | -3’097 | -4’076 |
|  | [-7’047 852] | [-8428 275] |
| Stripa tool costs | -3’589 | -3’586 |
|  | [-7’717 538] | [-7’714 541] |
| Time Recording costs | -3’608 | -3’567 |
|  | [-7’736 519] | [-7’695 560] |
| Drug costs | -3’823 | -3’353 |
|  | [-7’785 138] | [-7’687 980] |

Note: Results of the incremental cost obtained from GSEM models where one of the costs’ categories in turn (specified in the left column) varied. In the first column the specified cost category is decreased by 30%, while in the last column the specified cost category is increased by 30%. Local costs were expressed in Swiss Francs (CHF) using the PPP index. 95% confidence intervals in brackets.

*References*

1. SwissDRG <https://www.swissdrg.org/fr>. (Accessed 20 November 2019).

2. Swiss Federal Statistcal Office. Health - Pocket Statistics 2018. <https://www.bfs.admin.ch/bfs/fr/home/statistiques/sante/systeme-sante.assetdetail.7347549.html>. (Accessed May 2019). 2019.

3. Swiss Federal Office of Public Health. Spezialitätenliste. <http://www.spezialit>ätenliste.ch/. Accessed 2019-10-12. 2019.

4. Department of Health and Children. Case-mix measurement in Irish hospitals: A broad outline of the main features. Dublin, Ireland; 2003.

5. Healthcare Pricing Office. Admitted Price List 2020

<http://www.hpo.ie/abf/ABF2020AdmittedPatientPriceList.pdf>. (Accessed 15 September 2019).

6. Meirmans J. Nursing Homes Support Scheme Trends and Figures. Dublin: Irish Government Economic & Evaluation Service; 2017.

7. Health Sector. Consolidated salary scales in accordance with FEMPI 2015 and the Public Service Stability agreements 2013 - 2020. <https://www.hse.ie/eng/staff/resources/hr-circulars/hr-circular-020a-2018-amended-consolidated-salary-scales-1oct18.pdf>. 2018.

8. Cleemput I, Neyt M, Van De Sande S, Thiry N. Belgian guidelines for economic evaluations and budget impact analyses: second edition. KCE reports. 2012;183C.

9. Open Drug Database (ODDB). Accessed 2021-01-15.

10. National Institute for Health and Disability Insurance (NIHDI). <https://www.riziv.fgov.be/fr/themes/cout-remboursement/par-mutualite/prestations-individuelles/prix/Pages/default.aspx>. (Accessed 20 April 2020). 2020.

11. Yang Z, Zeng H, Xia R, Liu Q, Sun K, Zheng R, et al. Annual cost of illness of stomach and esophageal cancer patients in urban and rural areas in China: A multi-center study. Chinese Journal of Cancer Research. 2018;30(4):439.

12. Soley-Bori M, Ashworth M, Bisquera A, Dodhia H, Lynch R, Wang Y, et al. Impact of multimorbidity on healthcare costs and utilisation: a systematic review of the UK literature. Br J Gen Pract. 2021;71(702):e39-e46.

13. Zorginstituut Nederland. Richtlijn voor het uitvoeren van economische evaluaties in de gezondheidszorg. Diemen: Zorginstituut Nederland; 2016.

14. Kanters TA, Bouwmans CAM, van der Linden N, Tan SS, van Roijen LH. Update of the Dutch manual for costing studies in health care. PLOS ONE. 2017;12(11):1-11.

15. Kanavos P, Vandoros S, Irwin R, Nicod E, Casson M, Medical Technology Research Group (LSE Health), et al. DIfferences in costs of and access to pharmaceutical products in the EU. Brussels: European Parliament; 2011.

16. Gol-Montserrat J, del Burgo MLM, Quecedo L, del Llano JE. Analysis of Productivity Costs in Cancer: A Systematic Review. Global & Regional Health Technology Assessment. 2017;4(1):grhta.5000262.

17. Rubin DB. Multiple Imputation for Nonresponse in Surveys. New York: John Wiley and Sons; 1987.

18. Leurent B, Gomes M, Faria R, Morris S, Grieve R, Carpenter JR. Sensitivity Analysis for Not-at-Random Missing Data in Trial-Based Cost-Effectiveness Analysis: A Tutorial. PharmacoEconomics. 2018;36(8):889-901.
